# Complete genomic sequence and phylogenomics analysis of *Agrobacterium* strain AB2/73: a new *Rhizobium* species with a unique mega-Ti plasmid

**DOI:** 10.1186/s12866-021-02358-0

**Published:** 2021-10-28

**Authors:** Marjolein J. G. Hooykaas, Paul J. J. Hooykaas

**Affiliations:** Institute of Biology, Leiden University, Leiden, The Netherlands

**Keywords:** Agrobacterium, Crown gall, Ti plasmid, AB2/73, Limited host range, Opine, Phylogenomics, Whole-genome sequencing, Nanopore sequencing

## Abstract

**Background:**

The *Agrobacterium* strain AB2/73 has a unique host range for the induction of crown gall tumors, and contains an exceptionally large, over 500 kbp mega Ti plasmid. We used whole genome sequencing to fully characterize and comparatively analyze the complex genome of strain AB2/73, including its Ti plasmid and virulence factors.

**Results:**

We obtained a high-quality, full genomic sequence of AB2/73 by a combination of short-read Illumina sequencing and long-read Nanopore sequencing. The AB2/73 genome has a total size of 7,266,754 bp with 59.5% GC for which 7012 genes (6948 protein coding sequences) are predicted. Phylogenetic and comparative genomics analysis revealed that strain AB2/73 does not belong to the genus *Agrobacterium*, but to a new species in the genus *Rhizobium*, which is most related to *Rhizobium tropici*. In addition to the chromosome, the genome consists of 6 plasmids of which the largest two, of more than 1 Mbp, have chromid-like properties. The mega Ti plasmid is 605 kbp in size and contains two, one of which is incomplete, *repABC* replication units and thus appears to be a cointegrate consisting of about 175 kbp derived from an unknown Ti plasmid linked to 430 kbp from another large plasmid. In pTiAB2/73 we identified a complete set of virulence genes and two T-DNAs. Besides the previously described T-DNA we found a larger, second T-DNA containing a 6b-like *onc* gene and the *acs* gene for agrocinopine synthase. Also we identified two clusters of genes responsible for opine catabolism, including an *acc*-operon for agrocinopine degradation, and genes putatively involved in ridéopine catabolism. The plasmid also harbours *tzs*, *iaaM* and *iaaH* genes for the biosynthesis of the plant growth regulators cytokinin and auxin.

**Conclusions:**

The comparative genomics analysis of the high quality genome of strain AB2/73 provided insight into the unusual phylogeny and genetic composition of the limited host range *Agrobacterium* strain AB2/73. The description of its unique genomic composition and of all the virulence determinants in pTiAB2/73 will be an invaluable tool for further studies into the special host range properties of this bacterium.

**Supplementary Information:**

The online version contains supplementary material available at 10.1186/s12866-021-02358-0.

## Background

Bacteria of the genus *Agrobacterium* are the causal agents of the neoplastic plant diseases crown gall and hairy root [1]. The genus is part of the *Rhizobiaceae* family and species were initially defined based on their phytopathogenic properties: *A. tumefaciens* for crown gall tumor-inducing bacteria, *A. rhizogenes* for hairy root-inducing bacteria and *A. radiobacter* for avirulent strains. However, this classification appeared to be based on plasmid-determined properties, with Ti plasmids responsible for the induction of crown gall and Ri plasmids responsible for induction of hairy root. Regardless of the presence of any of these plasmids, *Agrobacterium* strains could be divided into three biovars based on phenotypic and metabolic characteristics [2]. Today, new taxonomic species and genera have been defined based on genomic relationships of these bacteria, which largely correspond to the previously defined biovars. For biovar 1 bacteria, the genus name *Agrobacterium* is kept, although more than 13 different genomovars (species) are now distinguished within this genus, each with specific ecological adaptations [3]. Bacteria of the genus *Agrobacterium* typically have both a circular and a linear chromosome [4]. To biovar 2 bacteria the species name *Rhizobium rhizogenes* was given and to biovar 3 bacteria the name *Allorhizobium vitis* [5]. These very different bacteria may nevertheless contain virtually identical Ti plasmids. More recently, four new tumorigenic strains isolated from blackberry galls were found to belong to the genus *Rhizobium*, but differ from *R. rhizogenes* and were given the species name *Rhizobium tumorigenes* [6].

Ti plasmids contain one or more T-DNA regions, sections that are transferred to plant cells during infection [1]. The T-DNA genes are expressed in the transformed plant cells and convert them into tumor cells, which eventually form a crown gall tumor on the infected plant. Responsible T-DNA genes often include genes involved in the biosynthesis of auxin (*iaaM*, *iaaH*) and cytokinin (*ipt*), as well as genes such as 6b belonging to the phenotypic plasticity (*plast*) family that modulate plant growth in an unknown manner [7]. In crown galls, specific metabolites called opines are formed that can be specifically broken down by the infecting bacteria [8]. The formation of the opines is catalyzed by opine synthases encoded by the T-DNA. The opine catabolic genes are also encoded on the Ti plasmid, but in a region outside of the T-DNA. The Ti plasmids are often classified by the specific opine(s) formed in the tumor, e.g. octopine, nopaline, succinamopine Ti plasmids.

The T-DNA is transferred in a single stranded form (T-strand) into plant cells by a transfer system encoded by the virulence region on the Ti plasmid and which is evolutionary related to the bacterial *incP* plasmid broad host range conjugation system [9, 10]. The virulence genes involved are distributed over several operons including operon *virB1-11* encoding the Type IV secretion system (T4SS), required for T-DNA delivery. The *virD1-2* and *virC1-2* genes are necessary for DNA processing and T-strand formation, and *virD4* encodes the coupling protein that can bring T-strands to the T4SS. The genes in the virulence region are controlled by a 2-component system consisting of the histidine receptor kinase VirA and the transcriptional activator VirG, which is activated by small plant derived compounds such as acetosyringone [11]. During infection the bacterium delivers not only T-strands to plant cells, but also a series of effector proteins (VirE2, VirE3, VirD5, VirF) that facilitate transformation [12].

Ti plasmids are conjugative plasmids that can spread to other bacteria through a series of conjugative transfer genes (*tra, trb*) that are activated in the presence of a specific opine [13]. Thus conjugative transfer can be observed in tumors, where these opines are abundantly formed [14]. Like most plasmids in *Rhizobiaceae* bacteria, Ti plasmids have a *repABC* unit for replication [15, 16]. In addition, they have still other, uncharacterized genes for unknown metabolic and other properties [10].

Most Ti plasmids confer on their hosts the ability to form tumors on a wide variety of dicot plants. However, some *A. vitis* strains can form tumors only on the vine and a limited number of other plant species. These limited host range (LHR) strains have a Ti plasmid with rearrangements in the T-DNA. An *Agrobacterium* strain with an extremely limited host range, called AB2/73, was isolated from a plant called *Lippia canescens* [17]. This bacterium can induce tumors on *Lippia*, and on squash and pumpkin, on which, remarkably, the normal wide host range (WHR) strains do not induce tumors [18], and thus AB2/73 may be a preferred gene vector for these plants. The Ti plasmid of strain AB2/73 was identified after conjugation *in planta* to a Ti plasmid-cured avirulent *Agrobacterium* strain, which rendered this strain tumorigenic. Tumors were induced by this transconjugant only on the same few plant species as by AB2/73 itself, showing that the limited host range properties were determined by pTiAB2/73 [19]. Although most Ti plasmids are about 200 kbp in size, the Ti plasmid from strain AB2/73 was reported to be over 500 kbp in size [19]. Otten and Schmidt [20] used a *vir* probe and the T-DNA border repeat as a probe on a Southern blot with pTiAB2/73 plasmid DNA to identify the *vir*-region and the T-DNA of pTiAB2/73. The *vir*-region was partially sequenced, while the T-DNA, located in a 3.5 kbp segment, was completely sequenced. It was found to contain two genes: gene *lsn* encoding a protein related to nopaline synthase and a gene *lso* encoding a *rolB*-like Plast (phenotypic plasticity) protein involved in tumor formation [7]. The same authors could not find other T-regions with the same approach. However, it seems likely that an extra T-region with an agrocinopine synthase gene must be present in strain AB2/73, as agrocinopines have been detected in tumors induced by AB2/73 [18]. Such an unknown T-region may very well contain other *onc* genes, as strain AB2/73 is more oncogenic and has a slightly wider host range for tumor induction than a strain that only introduces the *lso* gene into transformed plant cells [20].

We have now completely sequenced the genome of strain AB2/73 and this unexpectedly revealed that strain AB2/73 neither belongs to the genus *Agrobacterium*, nor to any of the species *R. rhizogenes, R. tumorigenes* or *A. vitis*, but to a new, so far undescribed *Rhizobium* species. We found that the mega Ti plasmid of this strain has a size of 605 kbp. It has two *repABC* replication units and appears to be a cointegrate of an (incomplete) unknown Ti plasmid with an entirely different, large plasmid. In the Ti plasmid portion of about 175 kbp, we found a complete set of *vir* genes and two regions surrounded by border repeats (T-DNAs) including a novel T-DNA with a 6b-like *onc*-gene and an *acs* gene for agrocinopine synthase. In the remainder of the Ti plasmid, we found a complete virulence region, a region containing genes for the biosynthesis of the plant growth regulators auxin (*iaaM*, *iaaH*) and cytokinin (*tzs/ipt*) and two regions containing genes for opine catabolism. Genes (*tra, trb*) for class I quorum-regulated conjugative Ti transfer, which are invariably present in Ti plasmids, were absent from pTiAB2/73, but other conjugative genes were present in the “non-Ti” part of the plasmid. We have used the pTiAB2/73 sequence for a detailed comparative analysis with other Ti and Ri plasmids.

## Results

### General features of the genome of *Agrobacterium* (*Rhizobium*) strain AB2/73

**Fig. 1:**
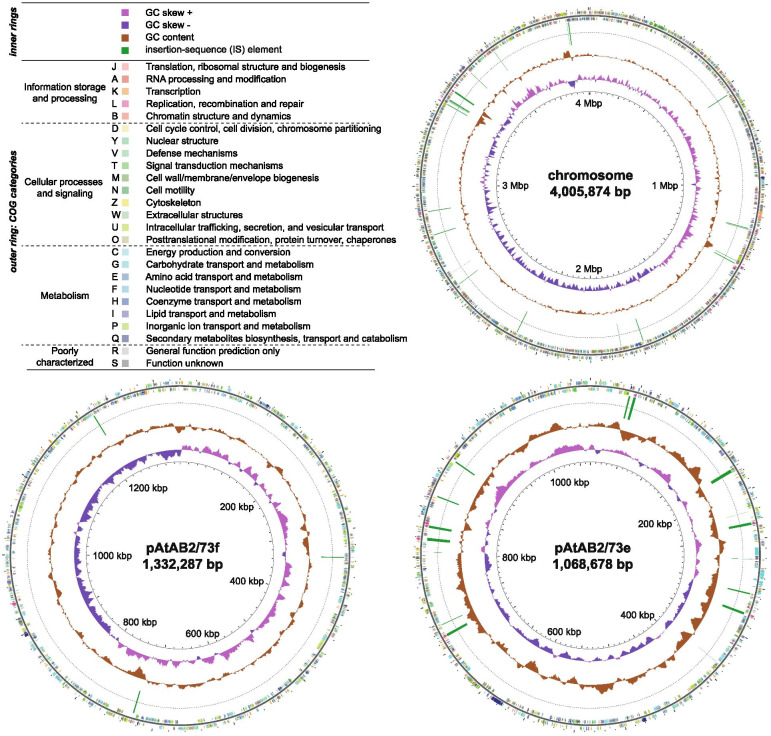
Circular representation of the largest three replicons of the *Rhizobium* sp. AB2/73 genome. The inner ring shows GC skew, which is defined as (G-C)/(G + C), where G is the number of guanines and C the number of cytosines. The parts where guanines are overrepresented are pink and the parts where guanines are underrepresented are purple. The next ring shows GC content. The ring with thin green rectangles indicates the locations of IS elements. The outer ring shows predicted protein coding genes colored by the COG functional category they were placed in

**Fig. 2:**
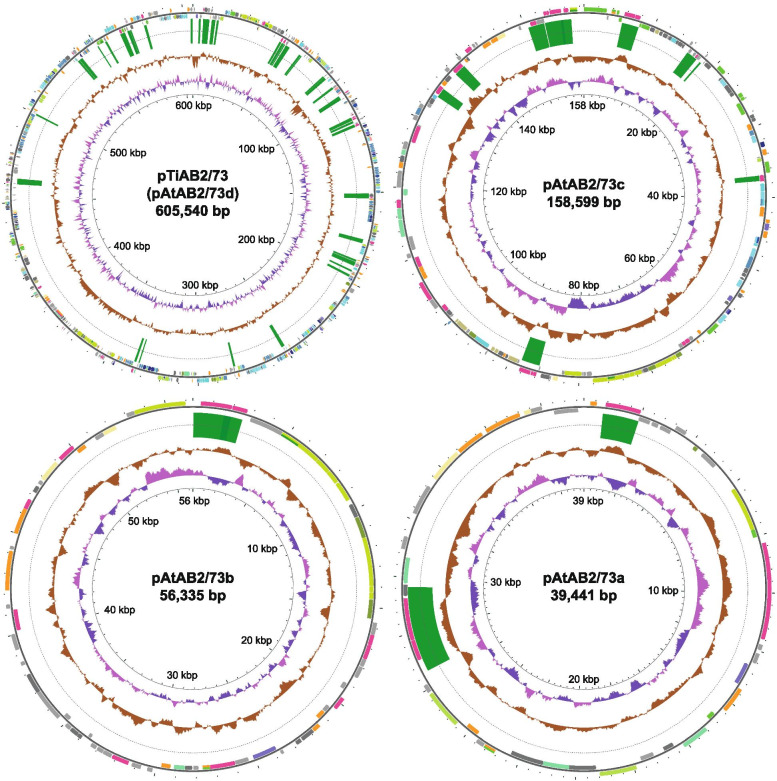
Circular representation of the smallest four replicons of the *Rhizobium* sp. AB2/73 genome. For a description of the rings and COG functional categories see Fig. 1

We obtained the full genomic sequence of AB2/73 by Unicycler hybrid assembly of short-reads from Illumina sequencing and long-reads from MinION Nanopore sequencing. The AB2/73 genome has a total size of 7,266,754 bp with 59.5% GC content for which 7012 genes (6948 protein coding sequences) are predicted. The chromosome has a size of 4,005,874 bp and contains the only three rDNA operons and all the 51 tRNA genes present in this bacterium. In addition to the chromosome, six DNA circles were identified with sizes of 1,332,287 bp, 1,068,678 bp, 605,540 bp, 158,599 bp, 56,335 bp and 39,441 bp. These represent the (mega) plasmids previously seen on gels and called pAtAB2/73f-pAtAB2/73a [19]. The chromosome is the only genetic element encoding a DnaA replication initiation protein; the other DNA circles all have a *repABC* system for replication as is most common for plasmids in the *Rhizobiaceae* family [15, 16]. For chromosome and plasmids, COG functional categories were assigned to predicted coding sequences, as can be seen on the circular maps in Figs. 1 and 2 and summarized for each of the circles in the bar charts in Fig. S1. The circular maps also show the GC content, GC skew, as well as the location of transposable elements. The smallest four replicons have a higher density of insertion-sequence (IS) elements, especially pAtAB2/73d (the Ti plasmid), which contains 47 IS elements (78 IS elements per million basepairs). While the GC content of the chromosome was 60.0%, this was only slightly lower at 59.3 and 59.7% for the two > 1 Mbp long plasmids, whereas it was 56.6-58.0% for the smaller plasmids. Both of the megabase-sized plasmids, like the primary chromosome, but unlike the smaller replicons, clearly have one part with mainly positive GC skew, whereas the other half shows mainly negative GC skew (Figs. 1 and 2). Such bias in guanines/cytosines composition (GC skew) across both DNA strands is present in most bacterial chromosomes [21, 22]. This asymmetry is thought to (partially) develop over evolutionary time because of the mechanistic difference between leading and lagging strand synthesis during genome replication, whereby selection pressure leads to an enrichment of guanines in the leading strand. The AB2/73 megabase-sized plasmids thus appear to have been under such selective pressure long enough for this GC skew to develop. The similarity in GC content and GC skew to the primary chromosome suggests that these two > 1 Mbp long plasmids may be chromids, mega-plasmids developing into secondary chromosomes [23].

### Comparative genomics and phylogenetic relatedness

**Fig. 3:**
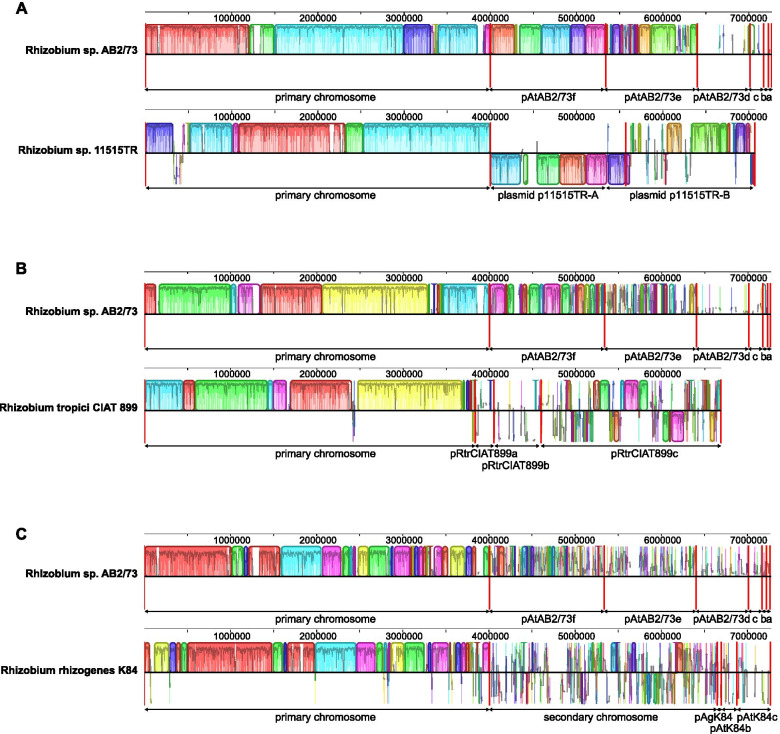
Pairwise whole genome alignments of AB2/73 to other *Rhizobium* species. The AB2/73 genome sequence was aligned to those of **A***Rhizobium* sp. 11515TR **B***Rhizobium tropici* CIAT 899 and **C***Rhizobium rhizogenes* K84 with progressiveMauve. Local collinear blocks are shown as blocks with the same color. Blocks below the centre line are aligned in reverse complementary orientation compared to the reference (top) sequence and blocks above the centre line are in forward orientation. Similarity profiles are shown inside the boxes. Vertical red lines, as well as the ends of the arrows with the replicon names below, mark the ends of replicons

The strain AB2/73 was originally classified as a biotype 2 *Agrobacterium,* nowadays *Rhizobium rhizogenes* [19]. However, AB2/73 does not belong to the species *R. rhizogenes.* Its genome shares only 83.3% ANI and 25% dDDH with the two fully sequenced *R. rhizogenes* strains, K84 [24] and LBA9402 [25]. Neither does AB2/73 belong to the recently described species *Rhizobium tumorigenes* [6] with which it shares only 79.3% ANI and 20.9% % dDDH. The genome shows more similarity to that of *Rhizobium tropici* strain CIAT899 (89.8% ANI, dDDH 38.6%), that of *Rhizobium* sp. strain 11515TR (93.2% ANI, dDDH 50%), and especially that of the partially sequenced *Rhizobium* sp. strain YK2 (99.5% ANI, 96.7% dDDH). Strain AB2/73 thus belongs together with strain YK2 to a new *Rhizobium* species. A species tree showing the relationship of AB2/73 to other *Rhizobium* and *Agrobacterium* species is shown in Fig. S2. Whereas *R. rhizogenes* and *R. tropici* have only two replicons of > 1 Mbp, the *Rhizobium* sp. 11515TR genome, like AB2/73, has three replicons of > 1 Mbp: in addition to its primary chromosome of 4,003,789 bp, it also has two replicons of around 1.5 Mbp. Not only the chromosome, but also these last two replicons show a significant overall similarity to replicons 2 and 3 of AB2/73 (Fig. 3).

**Fig. 4:**
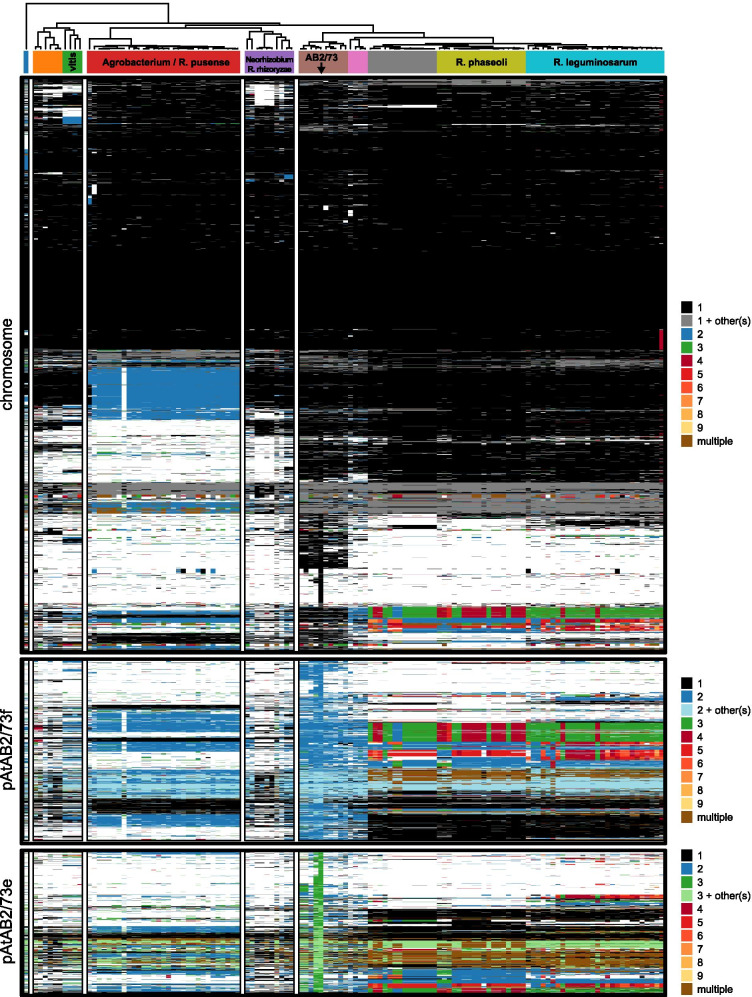
Heatmap showing conservation of AB2/73 genes across *Rhizobium/Agrobacterium* genomes. The proteins of 125 *Rhizobium* and *Agrobacterium* species including AB2/73 were clustered with OrthoFinder as described in the Methods section. The presence of homologs of the AB2/73 proteins in other genomes is shown for the three largest replicons: the primary chromosome, pAtAB2/73f and pAtAB2/73e. Each row represents an orthogroup (gene) and each column a genome. The columns are ordered by the leaves of the concatenated protein-alignments-based species tree (Fig. S2). The heatmap is vertically split up into five sections based on the structure of the species tree shown at the top and the segments in the color bar indicate a further division of the tree into smaller subclades of related species/strains. The color bar colors are also used in the species tree to show these same clades. The heatmaps consist of small blocks of which the colors represent the relative size-rank of the replicon the gene is located on, as shown in the legend, where 1 represents the largest replicon. Thus, genes present only on the primary chromosome (always the largest replicon), are shown as a small black box, genes present on the semi-largest replicon (in *A. tumefaciens* and *A. fabrum* e.g. the linear chromosome) are shown in blue etc. The numbers of replicons per genome and their size is shown in Fig. S6. NB, multiple homologous genes of the same genome may be assigned to the same orthogroup. These genes are either categorized as replicon x + other (s), or “multiple”. For example, in the heatmaps primary chromosome section, genes present both on the primary chromosome and on another replicon are shown in grey, while genes present on multiple replicons but not on the primary chromosome are shown in brown

Over evolutionary time genes have been transferred from chromosome to chromids (and back) in the *Rhizobiales* and thus the location of genes in a bacterium can also provide evolutionary and phylogenetic insight [24]. To show the exchange of genes between the primary chromosomes and smaller replicons, gene presence-absence heatmaps of the three largest AB2/73 replicons were constructed, which are shown in overview in Fig. 4, and with gene names added, for each of the three largest replicons separately in Figs. S3, S4 and S5. These figures show not only the presence-absence, but also by their color the replicon locations of homologs of the AB2/73 genes in the genomes of other members of the *Rhizobiaceae* family with varying degrees of genome similarity to AB2/73. The species analyzed are listed in Table S1, while an overview of the numbers and sizes of replicons per genome is shown in Fig. S6. The heatmaps reveal patterns which are largely in agreement with the species tree, which is based on concatenated (chromosomal) protein alignments (shown above the heatmaps and in Fig. S2). That is, genomes which are part of the same clade appear to show (mostly) similar patterns of gene presence and/or location.

The heatmap of the chromosomal genes (Fig. 4, Fig. S3) shows many (core) genes which are present in all genomes, but there are also clusters of genes which are only present in the *Rhizobium* species in the right half of the tree (including *R. leguminosarum, R. phaseoli, R etli, R. rhizogenes*)*,* but absent in the species in the left half of the tree (including *Agrobacterium, Allorhizobium vitis* and *Neorhizobium*). Finally, a number of chromosomal gene clusters are shared only with the species within the clade including *R. tropici*, *R. rhizogenes*, *R. lusitanum* and *R. jaguaris* (brown segment of the color bar) and some other gene clusters, including two representing putative integrated prophages, are unique for AB2/73. The heatmap also reveals the many chromosomal genes which in *Agrobacterium* (red segment of color bar) have moved to the linear chromosome in the course of evolution (replicon 2, blue). Some genes, such as the *hut* and *panCB* genes, are only located on the primary chromosome in the case of AB2/73 and its closest relatives (brown segment of the color bar), but not in the other bacterial strains analyzed. This is probably as a result of a retro-transfer process, after initially having been present on the chromid of a *Rhizobiales* ancestor [24, 26]. The clade including AB2/73, *R. tropici, R. rhizogenes, R. lusitanum* and *R. jaguaris* (brown segment of the color bar) shares with the *Agrobacterium* clade the location of the *cyo* genes (cytochrome-o-ubiquinol-oxidase-subunits) on the primary chromosome, in contrast to the other *Rhizobium* sp. in the right half of the tree which harbor these genes on plasmids.

The second-largest replicon pAtAB2/73f, with a size of 1332 kbp, contains the cell division genes *minCDE* and the *pca* genes for protocatechuate degradation, which were transferred from chromosome to chromid before the radiation of the rhizobia [24]. pAtAB2/73f also carries genes for DNA recombination by end-joining (two genes for both Ku and LigD each), and for degradation of erythritol and thus growth on erythritol. There are fewer genes on pAtAB2/73f which are uniquely shared by the clade including AB2/73, *R. tropici,* and *R. rhizogenes* (brown segment of the color bar) as compared to chromosomal genes. However, the clade still shows a distinct color pattern in the heatmap. The neighboring clade containing *Rhizobium grahamii* BG7 and *Rhizobium gallicum* IE4872 (pink segment in the color bar) shows a similar color pattern in the pAtAB2/73f heatmap section and thus shares many of the pAtAB2/73f genes on their secondary replicons, which are very large in these bacteria. However, these genes are mostly either on the primary chromosome or on plasmids size-ranked third or fourth (still hundreds in kb in size) in the other *Rhizobium* clades (*R. etli, R. phaseoli, and R. leguminosarum*). Approximately half of the pAtAB2/73f genes are also present in *Agrobacterium* species, located either on the linear chromosome (also size-ranked second) or on their primary chromosome. On pAtAB2/73f there are a number of genes which are present in all five bacterial strains/species in the AB2/73 - *R. tropici* subclade (left half of the brown segment in the color bar), but absent in its sister subclade (*R. rhizogenes, R. lusitanum* and *R. jaguaris*), e.g. *paaF - paaZ* (a phenylacetate metabolic gene cluster).

In the pAtAB2/73e heatmap, the clade with AB2/73, *R. tropici, and R. rhizogenes* lacks a clear signature. However, most genes in pAtAB2/73e are shared with the third replicon of *Rhizobium* sp. 11515TR, in line with the whole genome alignment (Fig. 3), and also a significant portion is shared with replicons ranked third in *Rhizobium* sp. CCGE531 and CCGE532, which are also part of the AB2/73 - *R. tropici* subclade (left half of the brown segment). In contrast, however, the homologs of pAtAB2/73e genes are located on the relatively large, second-largest replicon in the *R. tropici* CIAT 899 genome. Perhaps a part of the third replicon has been joined with the second replicon in *R. tropici*. Homologs of the pAtAB2/73e genes are located both on primary chromosomes, large replicons/chromids and smaller plasmids or combinations of replicons in more distantly related *Rhizobium* genomes.

Overall, the data suggest that a common ancestor of AB2/73, *Rhizobium* sp. 11515TR, *R. tropici* and *Rhizobium* spp. CCGE531, CCGE532 harbored large pAtAB2/73f- and pAtAB2/73e-like replicons and that even though global collinearity has been lost (Fig. 3), the gene functions have, for a significant part, been maintained on the large replicons of its descendants. This conservation fits well with their other chromid-like features (GC content and GC skew similar to primary chromosome).

### Replicons pAtAB2/73a, pAtAB2/73b, and pAtAB2/73c

Plasmids pAtAB2/73b and pAtAB2/73c are probably conjugative because they contain the necessary conjugative transfer genes, while the smallest 39 kbp plasmid pAtAB2/73a contains genes for mobilization proteins and for a coupling factor, but lacks the genes for a Type IV secretion system (T4SS). Thus the last plasmid may still be transmitted to new hosts using the T4SS encoded by one of the other plasmids. Plasmids with high similarity to pAtAB2/73a and pAtAB2/73c were not found in the genomes of any of the agrobacteria/rhizobia sequenced so far, but small portions of about 10% of these plasmids were found in several plasmids in bacteria of the *Rhizobiaceae* family. However, plasmids were found with high overall similarity to pAtAB2/73b. For example, the *A. vitis* plasmid pAtS4a, which is somewhat larger than pAtAB2/73b, shows 92% nucleotide identity in the shared segments (74% coverage). Remarkably, in the 158 kbp plasmid pAtAB2/73c there are genes for a restriction-modification system, and for DNA metabolism such as *dnaQ* (proofreading exonuclease), *helD* (helicase IV) and *gyrA* (topoisomerase IIA); in the 56 kbp plasmid pAtAB2/73b genes encoding LigD and UmuCD (involved in SOS repair) are found.

### Plasmid pAtAB2/73d: the mega-Ti plasmid of AB2/73

**Fig. 5:**
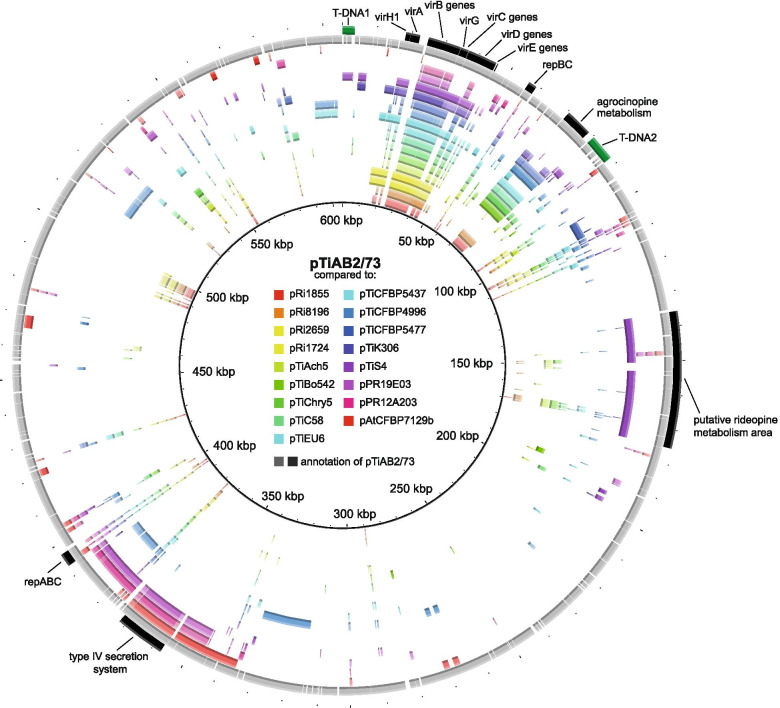
Sequence comparison between pTiAB2/73 and various Ti, Ri, Sym and At plasmids. BLASTn hits are shown in concentric rings, from inner to outer ring: agropine Ri plasmid pRi1855 (accession CP044124), mannopine Ri plasmid pRi8196 (accession NZ_JAAMDI010000041.1), cucumopine Ri plasmid pRi2659 (accession NZ_CP019703), mikimopine Ri plasmid pRi1724 (accession NC_002575), octopine Ti plasmid pTiAch5 (accession NZ_CP007228), agropine Ti plasmid pTiBo542 (accession NC_010929), chrysopine Ti plasmid pTiChry5 (accession KX388536), nopaline Ti plasmid pTiC58 (accession NC_003065), succinamopine Ti plasmid pTiEU6 (accession KX388535), Ti plasmid pTiCFBP5473 (accession NZ_CP039694, from *Agrobacterium larrymoorei*), Ti plasmid pTiCFBP4996 (accession NZ_CM016551, mega Ti plasmid), Ti plasmid pTiCFBP5477 (accession NZ_CM016547, from *A. larrymoorei*), Ti plasmid pTiK306 (accession NZ_JABFNP010000003, from *A. vitis*), vitopine/ridéopine Ti plasmid pTiS4 (NC_011982, from *A. vitis*), Sym plasmid pPR19E03 (accession NZ_CP054030, from *Rhizobium hidalgonense* strain JKLM 19E), Sym plasmid pPR12A203 (accession NZ_CP054024, from *Rhizobium indicum* strain JKLM 12A2), plasmid pAtCFBP7129b (accession NZ_CP039925). Color intensity indicates the degree of sequence similarity. The second-last ring shows predicted coding sequences and in the outermost ring a number of areas and genes are indicated

A plasmid estimated in size at the time to be about 500 kbp (pAtAB2/73d) was identified as the Ti plasmid, as this plasmid was seen in the recipient upon transfer of virulence [19]. We found that this plasmid corresponds to the 605,540 bp plasmid, which we identified by sequencing. A total of 575 protein coding sequences were found with an average size of 904 bp. No genes encoding transfer RNA (tRNA), or ribosomal RNA (rRNA) were found in pTiAB2/73. The plasmid backbone of pTiAB2/73 is very different from other Ti plasmids (Fig. 5). Compared to other Ti and Ri plasmids, it can be seen that Ti-like sequences together form an almost continuous segment of about 175 kbp. Beyond this Ti-like segment, a large 430 kbp region is present with genes of largely unknown function with no counterpart in well-characterized Ti plasmids (Fig. 5). Within this 430 kbp segment a *repABC* operon is located with a *repC* replicator gene which is only distantly related to that of other Ti plasmids so far characterized (Fig. S7). However, a second truncated *repABC* operon is present in the Ti-like segment with a *repC* gene that is related to that of other Ti plasmids (Fig. S7). Thus the pTiAB2/73 plasmid was probably formed by co-integration of two plasmids, an unknown Ti-plasmid and an entirely different plasmid. The Ti-like *repABC* replicator was (partially) inactivated due to rearrangements that may have been caused by the insertion of a number of transposable elements. As a result, only a small portion of *repA* is still present in a fusion with a portion of *repB*, as can be seen when comparing this truncated *repABC* operon to that of plasmid pAtCFBP4996a, to which the *repA* portion is most similar (Fig. S8). Ti and Ri plasmids described so far all possess a class I quorum-regulated conjugative transfer system with highly conserved conjugation genes located in whole or in part directly adjacent to the *repABC* genes [27]. However, in the Ti-like segment of pTiAB2/73, these highly conserved genes are not present next to the truncated *repABC* unit, nor elsewhere in the plasmid. Such a quorum-driven conjugation system may have been lost during or after the cointegration event. Unrelated *trb/virB* genes for a Type IV secretion system (T4SS), a coupling protein and a protein with a putative relaxase/mobilization nuclease domain are present in the “non-Ti” portion of the plasmid. As shown for *trbB/virB11* (Fig. S9) as an example, the T4SS genes found in the non-Ti part of pTiAB2/73 do not cluster with the *trb* genes of Ti plasmids, nor with the Ti *virB* genes, but similar genes can be found in a large number of *Rhizobium* (Sym) plasmids (Fig. S9). Apart from the T4SS genes, the 430 kbp large non-Ti portion of pTiAB2/73 contains mainly metabolic genes (Fig. 2) that have no counterpart in Ti plasmids or the *Rhizobium* (Sym) plasmids mentioned above (Fig. 5). It became apparent that pTiAB2/73 and in particular the Ti part, contains a large number of transposable elements (TEs, Fig. 2). One consequence of their action was truncation of the pTi-like *repABC* operon, but they may also have been responsible for gross rearrangements including the loss of pTi-like *tra/trb* conjugation genes.

**Fig. 6:**
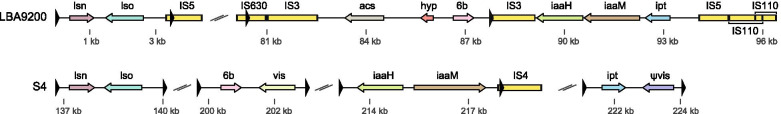
Overview of genes in the T-DNA regions of pTiAB2/73 and pTiS4. The smallest T-DNA of pTiAB2/73 is also present in pTiS4, while the other T-DNA is not conserved. IS elements including predicted terminal repeats are shown as yellow blocks. The other rectangles are predicted protein-coding genes, where blocks in the same color show similarity. Black triangles indicate the locations of the T-DNA border sequences. The part right of the right border, with the *iaaH*, *iaaM* and *ipt* genes, is thus located outside of the second T-DNA but is shown to indicate the proximity of these genes (which are usually located within the T-DNAs of other Ti plasmids, such as pTiS4)

We found four sequences which differed at most 1 nucleotide from the left and right T-DNA border consensus sequences (Table S2) in pTiAB2/73. One set surrounded the previously identified T-region (T-DNA1) of 3.5 kbp with the genes *lsn* encoding an opine synthase and *lso*, a *rolB*-like *plast* gene contributing to tumor formation [20]. It is unknown which opine is formed by the enzyme encoded by *lsn*. However, by protein sequence alignments we found that *A. vitis* plasmid pTiS4 harbors a gene highly similar (90.8% identical at protein level) to *lsn* in a fourth T-DNA (initially only three T-DNAs were reported in pTiS4 [28]). This T-DNA is very similar to T-DNA1 of pTiAB2/73 since both solely harbor a *nos/lsn*-like gene and a (homologous) *rolB*-like *plast* oncogene (Fig. 6; Fig. S10). It is known that in tumors induced by strain S4 the opines vitopine and ridéopine are produced [29]. While T-DNA2 of pTiS4 lacks opine synthase genes, T-DNA1 and T-DNA3 of pTiS4 contain vitopine synthase genes [28]. By inference it can therefore be concluded that the *lsn* opine synthase gene in pTiAB2/73 T-DNA1 and pTiS4 T-DNA4 must be responsible for the biosynthesis of the opine ridéopine. The finding that the opine synthase encoded by *lsn* bears some similarity to nopaline synthase, is consistent with this hypothesis as nopaline and ridéopine are formed by a similar chemical reaction, the conjugation of α-ketoglutarate with arginine and putrescine, respectively.

The pTiAB2/73 plasmid shares a large region of approximately 40 kbp, exclusively with the *A. vitis* pTiS4 and pTi1771 plasmids (Fig. 5, Fig. S10). This area, which includes both putative transporter and dehydrogenase genes, may be involved in opine uptake and catabolism. The 40 kbp region shared by pTiS4, pTiAB2/73 and pTi1771 is located adjacent to T-DNA4 in pTiS4. It is tempting to speculate that this region may be involved in the degradation of ridéopine. Genes for a lactamase/hydantoinase are also present, consistent with the fact that ridéopine can spontaneously form a lactam derivative [29]. A second region with the signature of an opine catabolic gene cluster in pTiS4, which is located close to T-DNA3 of pTiS4, has no counterpart in pTiAB2/73 or pTi1771 and is therefore likely involved in the degradation of vitopine.

The other T-region (T-DNA2) in pTiAB2/73 is 7.4 kbp in size and contains the gene for agrocinopine synthase (*acs*) and a gene related to the 6b oncogene. The gene content of the pTiAB2/73 T-DNAs is shown schematically in Fig. 6. The composition of T-DNA2 is unique to AB2/73 and differs from all the T-DNAs so far identified [30]. The *acs* gene most closely resembles the *acs* genes present in agropine-type pTiBo542 and chrysopine-type pTiChry5, while the 6b gene is most similar to that present in nopaline Ti plasmids such as pTiKerr108 [31]. The presence of an *acs* gene in pTiAB2/73 was not unexpected as agrocinopines had previously been detected in tumors induced by this strain [18].

The presence of the *acs* gene for agrocinopine synthase in one of the T-DNAs is accompanied by an agrocinopine catabolic operon with genes *accR* and an *accA-accG* operon in the region between the Ti-like *repC* and T-DNA2 (Fig. 5). The presence of these genes explains why strains containing pTiAB2/73 are sensitive to agrocin84 as previously established [19]. The catabolic *acc* genes of pTiAB2/73 are very similar to those of all other Ri and Ti plasmids conferring agrocinopine catabolism. However, the *accR* regulator is more similar to that of the Ti plasmids, than to that of the Ri plasmids.

Surprisingly, just downstream of the T-DNA2 right border *iaaM* and *iaaH* genes for bacterial auxin biosynthesis and an *ipt/tzs* gene for bacterial cytokinin biosynthesis are located (Fig. 6). These genes were previously not picked up when the plasmid was probed with T-DNA *iaaH*, *iaaM* and *ipt* genes [19]. This can be explained by our finding that these genes are only distantly related to the T-DNA genes (Figs. S11, S12 and S13). Their presence in pTiAB2/73 may contribute to the bacterial virulence of strain AB2/73, as in phytopathogenic bacteria such as *Pseudomonas savastanoi* [32].

The virulence operon was found to be located between the right border of T-DNA1 and the transposable elements that inactivated the *repABC* operon. At the right border end it starts with a *virH1* gene followed by *virA*, *virB1-11*, *virG*, *virC1-2*, *virD1-5* and ending with the *virE1-3* genes. The virulence genes are most related to those present in *A. vitis* Ti plasmids (e.g. pTiS4 and pTiK306), in cucumopine (pRi2659) and mikimopine (pRi1724) Ri plasmids and in *A. larrymoorei* pTiCFBP5473 (Fig. S14; Table S3). While most of the pTiAB2/73 *vir*-genes showed high sequence identity with the *vir*-genes from these plasmids, the *virE1-3* genes were much less conserved (Table S3). It was noteworthy that the *virA* gene of pTiAB2/73 lacks a *vir*-box and thus is not inducible by plant phenolics like that of *A. vitis* Ti plasmids and Ri plasmids. Lack of such *vir*-box in front of *virA* contributes to a host range restriction [33].

## Discussion

Comparative bioinformatics analysis of the genome of *Agrobacterium* strain AB2/73 revealed that this strain does not belong to any of the classic tumor-inducing species, the *Agrobacterium tumefaciens* species group, *Rhizobium rhizogenes* or *Allorhizobium vitis*. Neither does strain AB2/73 belong to *Neorhizobium* sp. [34] or *Rhizobium tumorigenes* [6], new species with crown gall tumor-inducing bacteria. Strain AB2/73 rather belongs to a new bacterial species together with some so far unnamed and largely uncharacterized *Rhizobium* strains. The most closely related, well-characterized species is *R. tropici* with which it shares about 90% average nucleotide identity. In the *Rhizobiales* genes have moved from the primary chromosome to megaplasmids and back over evolutionary time [24]. Also genes have been lost or acquired from the plasmid pool, eventually leading to adaptation to particular niches. Gene presence/absence heat maps of the chromosomes and largest plasmids (chromids) give an impression of the genome dynamics and underlying gene mobility and also give an indication about genomic relationships in addition to the more classic nucleotide identity scores. In this way a subclade of rhizobia became apparent, containing *R. rhizogenes*, but also *R. tropici* and AB2/73 that is distinct from other rhizobia such as *R. leguminosarum* and *R. etli*. Strain AB2/73 differs in genomic makeup from *R. rhizogenes* and *R. tropici* in having two chromids instead of one.

That several species of the *Rhizobiaceae* family can become tumorigenic after receipt of a Ti plasmid is already known for a long time. Introduction of a Ti plasmid into *Rhizobium trifolii*, *R. leguminosarum* and *R. phaseoli* [35, 36] and also into the even more distantly related *Phyllobacterium myrsinacearum* [37] rendered these bacteria tumorigenic. However, tumors formed by these bacteria were often smaller than those induced by the *Agrobacterium* parental strain, probably because of a lack of co-evolution of these species with the Ti plasmid, and in some cases a recipient *Rhizobium* such as *Sinorhizobium meliloti* remained avirulent after receipt of a Ti plasmid [38]. Nevertheless, *S. meliloti*, *Sinorhizobium fredii*, *Mesorhizobium loti* and *Ensifer adhaerens* were later developed as gene vectors for plants by introducing a disarmed Ti plasmid and a binary vector into these bacteria [39, 40].

Sequencing of *Rhizobium etli* strain CFN42 revealed interestingly that one of its plasmids, p42a, naturally contains a full set of Ti-like virulence genes, [41] and subsequent introduction of a binary vector converted this strain in a gene vector for plants [42]. Plasmid p42a was reported to lack a T-DNA, but nevertheless may be derived from a Ti plasmid. In agreement with this hypothesis we found five sequences which differed at most 1 nucleotide from the left and right T-DNA border consensus sequences (Table S4). Between border repeat 4 and 5 genes are present including a gene annotated as an octopine/nopaline dehydrogenase, which may encode an opine synthase expressed in transformed plant cells. During the preparation of our manuscript the presence of putative border repeat 4 and an *ocs*-like gene in p42a was reported by Otten [30]. Therefore, it seems that a Ti plasmid transferred into this *R. etli* strain may have been inactivated over time by deletion of much of its T-DNAs suggesting that this bacterium may not be adapted well enough to compete with agrobacteria in the tumor niche. Also one should therefore be cautious in using this strain as a gene vector, as the segments of the p42a plasmid surrounded by border repeats may also end up in the transgenic plants besides the binary vector T-DNA selected.

Our finding of two sets (one intact, one truncated) of *repABC* units suggests that pTiAB2/73 was formed by co-integration of two plasmids, a Ti plasmid and a much larger other plasmid. The Ti plasmid part contains relatively many transposable elements, which probably have caused rearrangements including deletion of some parts of the original Ti plasmid. For example, the Ti *repABC* operon is truncated and the Ti conjugation genes are all missing. Plasmid pTiAB2/73 is possibly still conjugative by a set of conjugation genes present in the non-Ti part. When AB2/73 was coinoculated in plants with a Ti plasmid cured recipient strain, a few transconjugants were obtained that had acquired the Ti plasmid [19]. When analyzed for plasmid content they had acquired not only pTiAB2/73, but also either pAtAB2/73b or pAtAB2/73c. Thus transfer may have been mediated by the putative conjugative system of pAB2/73 itself or by one of these plasmids, which we found to contain a full set of conjugative genes. As observed in other bacteria, transfer can then occur after cointegration of these plasmids in the donor, followed by transfer and resolution of the cointegrate in the recipient. Cointegration can be mediated by a transposase or an integrase present in one of the plasmids, or by the presence of homologous sequences shared by pTiAB2/73 and the conjugative plasmids [43, 44].

Previously only one T-DNA could be discovered in the pTiAB2/73 by using the border repeat as a probe on a Southern blot with the Ti plasmid DNA [20], but we have identified a second T-DNA (T-DNA2) by using the border repeat as a query on the whole genome sequence. This revealed in total four border repeats in pTiAB2/73, surrounding the two T-DNAs. The presence of a second T-DNA with a 6b *onc* gene may now explain why AB2/73 itself is more virulent on plants than a bacterium carrying the individual T-DNA1 with the single *lso onc* gene. The presence of T-DNA2 in pTiAB2/73 also explains the previously reported presence of agrocinopines in tumors induced by AB2/73 [18] as T-DNA2 contains the gene for agrocinopine synthase. T-DNA2 is unique to AB2/73, but T-DNA1 can also be found in the *A. vitis* pTiS4 plasmid ([30], our results), where it is present as the fourth T-DNA in addition to the three T-DNAs previously described [28].

While by Southern analysis it appeared that pTiAB2/73 lacked some detectable homology with the auxin (*iaaH, iaaM*) and cytokinin biosynthetic (*ipt*) T-DNA genes [19], remarkably, such genes were still present, but not in either of the two T-DNAs, but in the region next to T-DNA2. Not only was an *ipt* / *tzs*-like gene present there, but *iaaH* and *iaaM* genes were also found, which code for enzymes that together can form the auxin indole acetic acid (IAA). Such *iaa*-genes are common in other phytopathogenic bacteria such as *Pseudomonas savastanoi*, where the release of IAA from the bacteria can cause gall formation [32]. The *iaa*-genes in pTiAB2/73 may originate directly from another bacterium or were previously present in a T-DNA, but have been displaced outside the T-DNA by DNA rearrangements. When we aligned the encoded proteins and constructed phylogenetic trees, we found that the AB2/73 *iaaH* and *iaaM* encoded proteins are much closer related to those of phytopathogenic bacteria such as *Dickeya* (more than 60% identity) than to those encoded by T-DNAs (around 35-50% identity) (Figs. S11 and S12). It is likely therefore that the AB2/73 genes are not derived from a degraded T-DNA.

Strain AB2/73 was isolated from a crown gall on *Lippia canesens* and can induce tumors on very few other plants [17]. Interestingly, these plants include some squashes that cannot be transformed by standard agrobacteria [18]. The host range of strain AB2/73 is linked to the Ti plasmid; upon transfer to the Ti-cured laboratory strain C58, this strain became tumorigenic with the same limited host range as AB2/73 [19]. Previous research has been conducted on factors leading to a limited host range in *A. vitis* LHR Ti plasmids. The limited host range (LHR) in these strains could be extended by adding the *ipt* gene to the T-DNA of these strains, indicating that cytokinin production in the transformed plant cells is required for tumor proliferation in some host species [45, 46]. Thus, the absence of an *ipt* gene in the T-DNAs of pTiAB2/73 may be an important reason for its limited host range. However, Ti plasmid genes related to a limited host range have also been found in the virulence region [47]. The introduction of the *virA* gene from a WHR strain into an LHR *A. vitis* strain expanded the host range with *Kalanchoe daigremontiana* [47]. The requirement for a WHR Ti *virA* gene for tumor induction was due not so much to a different biological activity of the histidine kinase encoded by the WHR *virA* gene, but rather to a difference in expression due to the presence of a *vir*-box in the *virA* promoter [33]. The addition of such a *vir*-box to the promoter of the LHR Ti *virA* gene was sufficient to expand the host range for tumor induction with *Kalanchoe daigremontiana* [33]. The pTiAB2/73 plasmid has a complete virulence region with all essential *vir*-genes, but indeed, like *A. vitis* LHR strains, contains a *virA* gene without a *vir*-box. This can be seen in Table S5, which shows that a *vir* box in the *virA* promoter is present in almost all Ti and Ri plasmids, but is missing not only in the *A. vitis* Ti plasmids, but also in pTiAB2/73 and in the cucumopine and mikimopine Ri plasmids, the host range of which has not yet been described in detail. Why strain AB2/73 is tumorigenic on squashes in contrast to standard *Agrobacterium* strains could be related to the two *plast* oncogenes present in its T-DNAs. It was demonstrated that the 6b gene of octopine strains by itself can mediate tumorigenicity on certain *Kalanchoe* species [48], while 6b genes from different strains can have a wide range of oncogenic properties [7, 49]. It will be very interesting to test the oncogenic properties and host range of the AB2/73 *onc* genes.

## Conclusions

Whole genome sequencing followed by a detailed comparison of the genome of *‘Agrobacterium’* strain AB2/73 with that of other bacteria of the *Rhizobiaceae* family revealed that AB2/73 belongs to a new *Rhizobium* species, which is most related to *R. tropici* and some other, still unnamed rhizobia. In the rhizobia, genes have often migrated from chromosome to plasmids (and sometimes back) over evolutionary time. Heat maps made to compare the position of the genes on the chromosome and the two chromids of AB2/73 with those of their homologs in other rhizobia revealed unique patterns, distinguishing AB2/73 along with *R.tropici*, *R. jaguaris*, *R. lusitanum*, and *R. rhizogenes,* which together also form a separate clade in the species tree*,* from other bacteria of the *Rhizobiaceae* family. The virulence of AB2/73 turned out to be due to the presence of a unique 605 kbp mega-Ti plasmid, evolved from a cointegrate between a Ti plasmid and a much larger, unrelated plasmid. The description of its virulence genes, T-DNAs and opine catabolic genes will give direction to future studies regarding the unique host range of this bacterium.

## Methods

### Organism

*Agrobacterium* strain AB2/73 (LBA9200 in our collection) was obtained from prof. E. W. Nester (Seattle, USA). The bacterium was grown on TY medium (Difco tryptone 5 g/l, Difco yeast extract 3 g/l, CaCl2.6H2O 1.3 g/l) as described by Beringer [50]. The bacterium was tested for virulence by puncturing the plant stems of *Nicotiana glauca* with a sterile wooden toothpick that had been dipped into a colony of the bacterium.

### DNA sequencing

Genomic DNA was isolated using QIAGEN Genomic-tip gravity-flow columns. The genomic sequence of AB2/73 was determined using a combination of Illumina and Oxford Nanopore Technologies platforms. Nanopore sequencing was done in house, but Illumina sequencing was performed at the Leiden Genome Technology Center (LGTC) of the Leiden University Medical Center (Leiden, The Netherlands), where TruSeq DNA Libraries were sequenced on an Illumina HiSeq 2000 machine. The Oxford Nanopore sequencing library was generated with 200 ng DNA using the SQK-RBK004 Rapid Barcoding Kit. The library was pooled with another library, followed by in-house sequencing on a MinION flow cell (version R9.4.1). After basecalling with Albacore (version 2.3.4) the reads were demultiplexed (with Epi2me). The total yield for AB2/73 was 1,158,758 reads, totaling 5,160,617,317 bp (with quality > Q7), with a N50 read length of 8263 bp (710x coverage).

### Genome assembly and annotation

Nanopore reads were end-trimmed and filtered on average quality (>Q10) and length (> 10 kb) with NanoFilt (200-fold coverage after filtering). A total of 6,021,213,100-nucleotide paired-end Illumina reads were quality and adapter trimmed using Cutadapt (70-fold coverage). Hybrid assembly was performed using Unicycler version 0.4.7. The eighth contig, representing the bacteriophage PhiX genome sequence, spiked-in during Illumina library preparation, was removed. The complete genome sequence of AB2/73 was deposited in GenBank under accession numbers CP067071-CP067077. The raw reads are deposited in the Sequence Read Archive under accessions numbers SRR13775335, SRR13775336. Microbial Genome Atlas (MiGA), TypeMat, and NCBI Prok were used to identify related genomes from the NCBI RefSeq and Prokaryotic Genomes databases. TypeMat also provided estimations of genome completeness and contamination. Based on the presence of essential genes, the TypeMat estimate of genome completeness was 99.1%. ANI values were calculated with fastANI [51] and Digital DDH values with GGDC 2.1, whereby distances are inferred from identities/HSP (high scoring segment pairs) length for pairwise comparison of AB2/73 with other genomes [52]. The genome was annotated with NCBI Prokaryotic Genome Annotation Pipeline (PGAP [53]), as well as with eggnog-mapper v2 [54]. From the latter tool we obtained the assignments of proteins to Clusters of Orthologous Groups (COG) functional categories. ISEScan was used to annotate insertion sequences [55].

### Comparative genomics

Full genome alignments were performed with progressiveMauve [56]. Schematics of T-DNA regions, rep(A)BC operons and vir regions were generated with the R package genoplotR [57]. BLASTn comparisons between plasmids TiAB2/73 and pTiS4 and other plasmids were performed and visualized with BLAST Ring Image Generator (BRIG) [58]. Orthogroups of homologous proteins were inferred with OrthoFinder version 2.3.12 (all-versus-all DIAMOND search followed by MCL clustering) [59]. For comparisons of Ti and Ri plasmid encoded proteins, protein sequences from Ti and Ri plasmids (extracted from Genbank files) were clustered. For the heatmaps, proteins from 125 *Rhizobium* and *Agrobacterium* genomes (listed in Table S1), and an in house sequenced *Ochrobactrum* strain (named strain LBA8980) were clustered. Pseudogenes were excluded. Genomes were selected based on NCBI taxonomic classification in the *Rhizobium/Agrobacterium* group and level of completeness (complete genomes only). The genomes were from organisms of genera *Rhizobium, Neorhizobium, Agrobacterium and Allorhizobium (*since *Agrobacterium vitis* is nowadays officially called *Allorhizobium vitis).* The species tree in Fig. 2 was inferred with FastTree 2.1.10 based on a (trimmed) concatenated protein alignment of 1165 single-copy genes generated by OrthoFinder. The OrthoFinder orthogroups table was processed with R to generate tables summarizing the location of genes on the genomes’ replicons. Briefly, the protein identifiers were converted to (lists of) replicon ranks and this matrix was then merged to a table containing the AB2/73 locus tags (keeping only orthogroups present in the respective AB2/73 replicons). Single orthogroups are represented multiple times per matrix in case more than one AB2/73 gene has been assigned to it. For Fig. 4 the rows were clustered. A dissimilarity matrix was calculated based on Gower’s distances, followed by Ward clustering. The R package ComplexHeatmap was used for visualizing the heatmaps.

Protein alignments were performed with MAFFT version 7.471, L-INS-I method [60] and visualized with pyBoxshade. Percentage identities of Vir proteins in Table S3 were calculated with the R package seqinr (based on the multiple sequence alignments). The VirB11/TrbB maximum likelihood tree was inferred with FastTree 2.1.10 (after MAFFT protein alignment). The RepC maximum likelihood tree was inferred with IQ-TREE 2.0.3 (after MAFFT alignment and TrimAI trimming, JTT + R7 model). Trees were visualized with the Python library ETE toolkit (version 3) [61]. Part of the work was performed using the ALICE computer resources provided by Leiden University.

## Supplementary Information

**Additional file 1: Figure S1.** COG functional category classification of AB2/73 proteins. Shown is the number of proteins assigned to each COG functional category. Proteins falling into multiple categories were counted once for each category. On the right a description of the functional categories is given.

**Additional file 2: Figure S2.** Core genome phylogenetic tree. A phylogenetic tree of 125 *Agrobacterium, Allorhizobium, Rhizobium* and *Neorhizobium* species was inferred based on the concatenated protein alignments of 1165 single-copy genes. *Ochrobactrum* strain LBA8980 was used as an outgroup. The tree’s branch colors are used in the color bars of the heatmaps (Fig. 4, Figs. S3, S4 and S5) and in the replicon sizes bar chart (Fig. S6) to indicate corresponding genomes. NB, not every clade contains equally closely related bacterial species. The *R. leguminosarum/R. indicum* clade consists of rather closely related species (short branch lengths), in contrast to the orange and purple clades which have rather long branch lengths, but were not further subdivided in order to keep a clear view. The red clade, besides all *Agrobacterium* species, contains a number of *Rhizobium* species. This is not unexpected, since of these, *Rhizobium pusense* and *Rhizobium oryzihabitans* harbor (like *A. tumefaciens* and *A. fabrum*) a linear chromosome and *R. pusense* has also been named *Agrobacterium* genomospecies G2. *Rhizobium* sp. NIBRBAC000502774 shows a strange pattern in the heatmaps (Fig. 3, Figs. S3, S4 and S5). Detailed investigation of this genome assembly was beyond the scope of this study, but in the genome taxonomy database (GTDB) it is listed as an *Agrobacterium* species, with a CheckM completeness score of only 84.09%.

**Additional file 3: Table S1.** List of genomes used for analyses. Genomes are listed which were used for protein clustering, species tree construction and building the gene presence heatmaps.

**Additional file 4: Figure S3.** Heatmap showing conservation of AB2/73 chromosomal genes across *Rhizobiaceae* genomes. This figure is based on the same data as Fig. 4. However, in this larger version, rows were not clustered by similarity but ordered by the location of the genes in the AB2/73 chromosome.

**Additional file 5: Figure S4.** Heatmap showing conservation of pAtAB2/73f genes across *Rhizobiaceae* genomes. This figure is based on the same data as Fig. 4. However, in this larger version, rows were not clustered by similarity but ordered by the genes’ location in pAtAB2/73f.

**Additional file 6: Figure S5.** Heatmap showing conservation of pAtAB2/73e genes across *Rhizobiaceae* genomes. This figure is based on the same data as Fig. 4. However, in this larger version, rows were not clustered by similarity but ordered by the genes’ location in pAtAB2/73e.

**Additional file 7: Figure S6.** Stacked bar chart showing *Agrobacterium/Rhizobium* genome and replicon sizes. The text color and order of bars are as per the leaves of the species tree in Fig. S2. The replicon rank colors correspond to those used in the heatmaps.

**Additional file 8: Figure S7.** RepC phylogenetic tree. A maximum likelihood phylogenetic tree was constructed from the RepC protein sequences from 125 *Rhizobium* and *Agrobacterium* genomes. The resulting tree was midpoint rooted. The scale bar shows the number of amino acid changes per site. The tree was annotated with protein descriptions written as organism|replicon name|locus tag. AB2/73 proteins are printed in bold. Ti plasmid proteins are shown in red text, Ri plasmid proteins in orange text and Sym plasmid sequences in green. The black leaf nodes represent plasmid encoded proteins, whereas chromosomal proteins have blue leaf nodes (using the genome assemblies annotation as ‘chromosome’ or ‘plasmid’). Most (but not all) Ti plasmid RepC proteins are clustered together in a group indicated with red background. pTiAB2/73 harbors two *repC* genes. RepC encoded by the gene with locus tag I8E17_31105 (of the *repB’-repC* operon) lies in the group with the Ti plasmid RepC proteins, whereas the other RepC (of the *repABC* operon) groups with RepC proteins of some other non-Ti plasmids. RepC encoded by plasmids pAtAB2/73e and pAtAB2/73f cluster with RepC proteins from replicons of genomes to which AB2/73 is most related according to the species tree.

**Additional file 9: Figure S8.** pTiAB2/73 *repB* of the *repBC* operon is truncated. A comparison is shown of pTiAB2/73 *rep* genes (locus tags I8E17_31100, I8E17_31105) with the *repABC* operon of plasmid pAtCFBP4996a. The red ribbons show tBLASTx hits (e < 0.001), with color intensity indicating the degree of sequence identity (darker = higher similarity). The C-terminal part of I8E17_31100 (“*repB*”) is conserved, yet the N-terminus shows similarity to the N-terminus and an internal part of the *repA* gene of pAtCFBP4996a. The gene I8E17_31100 thus appears to be a truncated *repB* fused to fragments of *repA*.

**Additional file 10: Figure S9.** VirB11/TrbB phylogenetic tree. A maximum likelihood phylogenetic tree was constructed based on VirB11/TrbB protein sequences from 125 *Rhizobium* and *Agrobacterium* genomes. The tree was midpoint rooted. The scale bar shows the number of amino acid changes per site. The tree was annotated with protein descriptions written as organism|replicon name|locus tag. Ti plasmid proteins are shown in red text, Ri plasmid proteins in orange text and Sym plasmid sequences in green. AB2/73 proteins are printed in bold. TrbB sequences form a separate clade (the part with the yellow background). Generally, Ti and Ri plasmids have a *trb* region, so the TrbB clade includes Ti and Ri plasmid sequences, however a pTiAB2/73 TrbB homolog is lacking. The VirB11 sequences from the *vir* regions of Ti and Ri plasmids also cluster together (clade with very light blue background), including a pTiAB2/73 protein (locus tag I8E17_30995). The rest of the proteins in the tree (darker blue background) are also named ‘VirB11’ but these are not derived from Ti plasmid *virB* operons. The only exception is the second pTiAB2/73 VirB11 homolog. The gene encoding this protein (locus tag I8E17_32535) is located in the non-Ti-like part of pTiAB2/73. This protein clusters with sequences mostly encoded by *Rhizobium (indicum, leguminosarum, hidalgonense and etli*) Sym plasmids. Some of these Sym plasmids, like pTiAB2/73, have a second VirB11 homolog which is rather similar to vir region-containing VirB11 (green names in very light blue background). The similarity of pTiAB2/73 *vir* region *virB* genes and the second set of *virB*-like genes to two such Sym plasmids, as well as an example of a plasmid which only shows similarity of the *virB*-like operon, can be seen in Fig. 5 (rings pPR19E03, pPR12A203 and pAtCFBP7129b, respectively).

**Additional file 11: Table S2.** T-DNA border sequences found in pTiAB2/73.

**Additional file 12: Figure S10.** Ridéopine metabolism region presumably conserved across pTiS4, pTiAB2/73 and pTi1771. The sequence of pTiS4 (accession NC_011982) is compared to various Ti and Ri plasmid sequences. BLASTn hits are shown in concentric rings, from inner to outer ring: nopaline Ti plasmid pTiC58 (accession NC_003065), octopine Ti plasmid pTiAch5 (accession NZ_CP007228), agropine/succinamopine Ti plasmid pTiBo542 (accession NC_010929), agropine Ri plasmid pRi1855 (accession CP044124), mikimopine Ri plasmid pRi1724 (accession NC_002575), Ti plasmid pTi1771 (unknown opine type, unpublished, *A. vitis* strain NCPPB 1771) and pTiAB2/73 (accession CP067074). The DNA region adjacent to pTiS4 T-DNA region 4, and the nopaline-like opine synthase gene within T-DNA4 (*lsn*), are conserved (only) in pTiAB2/73 and pTi1771. Since *A. vitis* S4 produces the opines vitopine and ridéopine, and since the T-DNA regions 1-3 harbour either vitopine synthase or no opine synthase, the *lsn* gene in T-DNA4 likely encodes a ridéopine synthase, and the adjacent region likely harbors the genes required for ridéopine import and catabolism.

**Additional file 13: Figure S11.** IaaH/Tms2-like protein from pTiAB2/73 is more similar to non-T-DNA sequences than to T-DNA-encoded IaaH. Alignment of Indoleacetamide hydrolase/amidase sequences from pTiAB2/73, from two *Rhizobiaceae* family members (*Rhizobium tumorigenes* and *Allorhizobium vitis*, 87% identical), two betaproteobacterial amidases (*Dickeya dianthicola, Trinickia symbiotica*, 63% identical) and *iaaH/tms2* from the T-DNAs of the Ti plasmids of *A. tumefaciens* strains C58, Ach5 (LBA4213) and S4 (32-34% identical). Residues identical in the majority of sequences are shown with black shading, residues similar in the majority of sequences are shown with grey shading.

**Additional file 14: Figure S12.** IaaM/Tms1-like protein from pTiAB2/73 is more similar to non-T-DNA sequences than to T-DNA-encoded IaaM. Alignment of tryptophan 2-monooxygenase -like sequences from pTiAB2/73, from two *Mesorhizobium* species, from one *Rhizobium* species (~ 90% identical), one betaproteobacterial oxidoreductase (*Dickeya chrysanthemi*, 68% identical), and tryptophan 2-monooxygenases (*iaaM/tms1*) from the T-DNAs of the Ti plasmids of *A. tumefaciens* strains C58, Ach5 (LBA4213) and S4 (46-50% identical). Residues identical in the majority of sequences are shown with black shading, residues similar in the majority of sequences are shown with grey shading.

**Additional file 15: Figure S13.** pTiAB2/73 Ipt/Tzs-like protein shows strongest similarity to *tzs*-encoded isopentenyl transferases. Alignment of pTiAB2/73 *tzs*-like encoded protein to isopentenyl transferases from multiple Ri and Ti plasmids. Sequence identity versus pTiAB2/73 *tzs*-like varied from 44% (pTiS4 *ipt*) to 83% (pRi2659 *tzs*). Residues identical in the majority of sequences are shown with black shading, residues similar in the majority of sequences are shown with grey shading.

**Additional file 16: Figure S14.** Comparison between of a number of Ti plasmid *vir* regions. Comparison between the *vir* region of pTiAB2/73 with those of pRi2659 (cucumopine Ri plasmid), pTiCFBP5473 (Ti plasmid from *A. larrymoorei* strain CFBP5473) and pTiK306 (Ti plasmid from *A. vitis* strain K306). The tBLASTx hits (e < 0.001) are shown in red, with darker bands indicating higher degrees of similarity. Most *vir* genes are well conserved, but *virD3* and *virD5*, and *virE1*, *virE2* and *virE3* only to a lesser extent.

**Additional file 17: Table S3.** Vir protein similarities. Percentage identities between AB2/73 and other Ti plasmid Vir proteins.

**Additional file 18: Table S4.***R. etli* plasmid p42a contains T-DNA border-like sequences. This table shows the annotation of a part of *R. etli* plasmid p42a, with in addition to the gene annotation derived from accession NC_007762 the locations of a number of sequences which (almost) match T-DNA border consensus sequences. Also notable is the presence of a NAD/NADP-octopine/nopaline-dehydrogenase-family-protein between two of these borders and the large number of insertion sequences.

**Additional file 19: Table S5.** Table listing the presence or absence of a *vir*-box sequence (RYTNCANTTGNAAY) upstream of the *virA* gene.

## Data Availability

The complete genome sequence of AB2/73 was deposited in GenBank under accession numbers CP067071-CP067077. The raw reads are deposited in the Sequence Read Archive (https://www.nlm.nih.gov/sra) under accessions numbers SRR13775335, SRR13775336.

## Abbreviations

ANI: Average nucleotide identity
COG: Clusters of orthologous groups
dDDH: Digital DNA-DNA hybridization
HSP: High scoring segment pair
IS: Insertion sequence
LHR: Limited host range
TE: Transposable element
WHR: Wide host range
