# Supplementary material for: Complete genomic sequence and phylogenomics analysis of Agrobacterium strain AB2/73: a new Rhizobium species with a unique mega-Ti plasmid
Source: BMC Microbiol. 2021 Oct 28;21:295. doi: 10.1186/s12866-021-02358-0 (PMC8554961; doi:10.1186/s12866-021-02358-0)
Supplement: Supplementary file 1 — Additional file 1: Figure S1. COG functional category classification of AB2/73 proteins. Shown is the number of proteins assigned to each COG functional category. Proteins falling into multiple categories were counted once for each category. On the right a description of the functional categories is given. [file 12866_2021_2358_MOESM1_ESM.pdf]

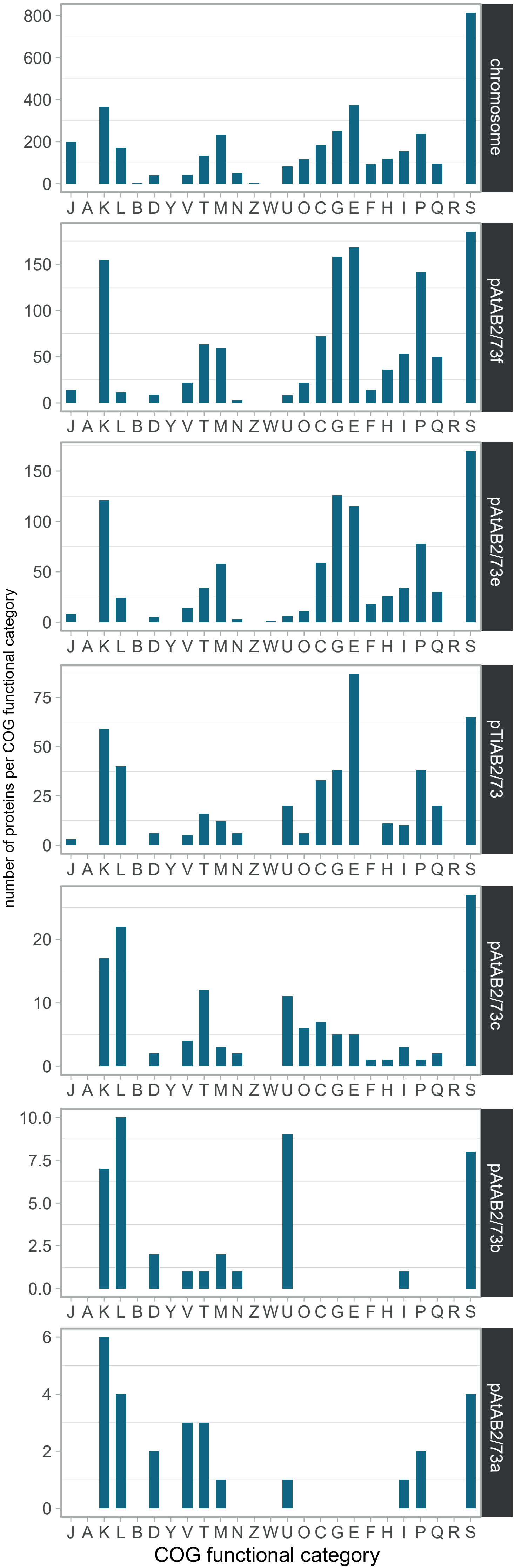

Class	Letter	Description
Information storage and processing	J	Translation, ribosomal structure and biogenesis
	A	RNA processing and modification
	K	Transcription
	L	Replication, recombination and repair
	B	Chromatin structure and dynamics
Cellular processes and signaling	D	Cell cycle control, cell division, chromosome partitioning
	Y	Nuclear structure
	V	Defense mechanisms
	T	Signal transduction mechanisms
	M	Cell wall/membrane/envelope biogenesis
	N	Cell motility
	Z	Cytoskeleton
	W	Extracellular structures
	U	Intracellular trafficking, secretion, and vesicular transport
	O	Posttranslational modification, protein turnover, chaperones
Metabolism	C	Energy production and conversion
	G	Carbohydrate transport and metabolism
	E	Amino acid transport and metabolism
	F	Nucleotide transport and metabolism
	H	Coenzyme transport and metabolism
	I	Lipid transport and metabolism
	P	Inorganic ion transport and metabolism
	Q	Secondary metabolites biosynthesis, transport and catabolism
Poorly characterized	R	General function prediction only
	S	Function unknown
