# Supplementary figures and images for: Complete genomic sequence and phylogenomics analysis of Agrobacterium strain AB2/73: a new Rhizobium species with a unique mega-Ti plasmid
Source: BMC Microbiol. 2021 Oct 28;21:295. doi: 10.1186/s12866-021-02358-0 (PMC8554961; doi:10.1186/s12866-021-02358-0)

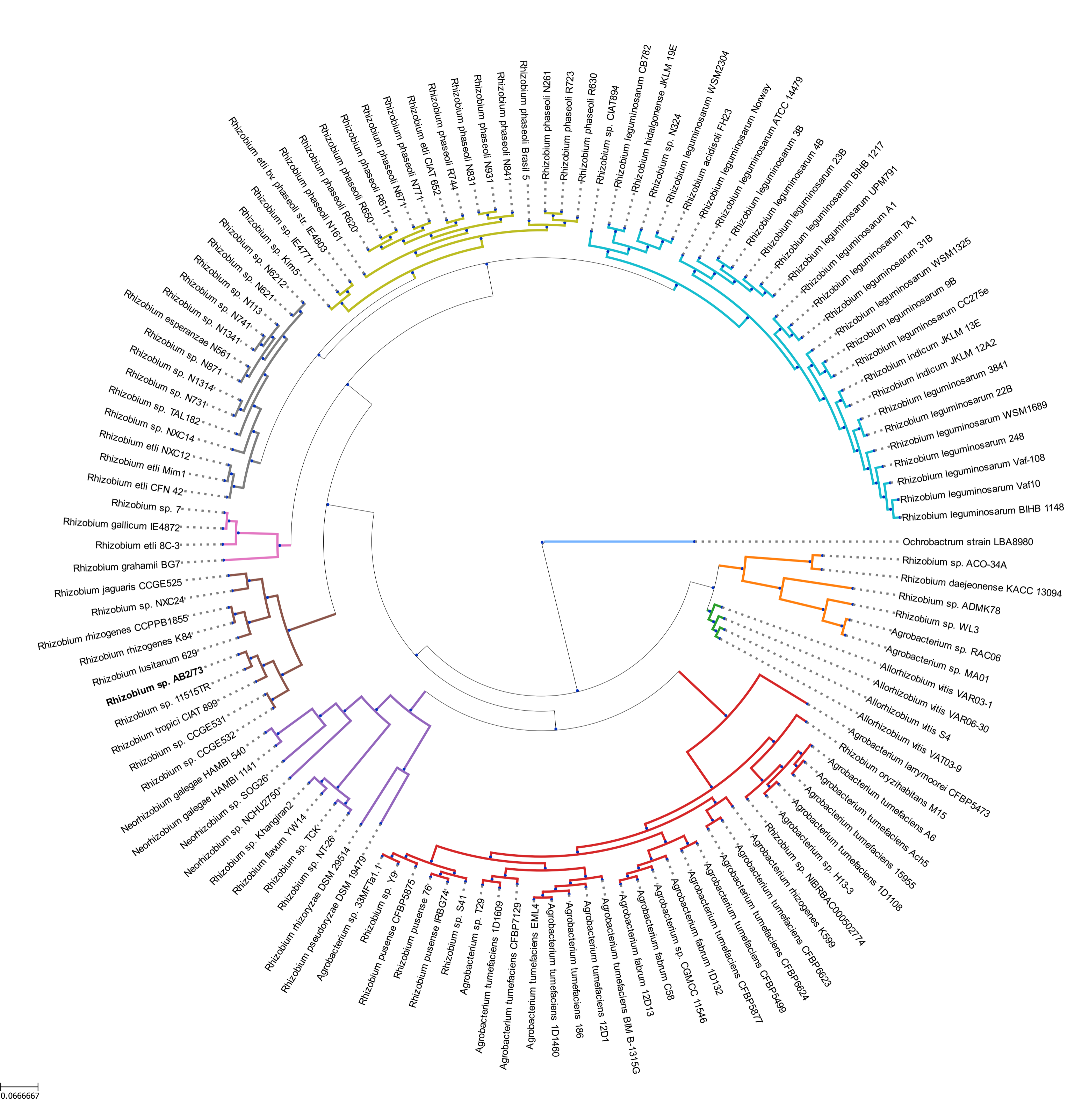

Supplement: Supplementary file 2 — Additional file 2: Figure S2. Core genome phylogenetic tree. A phylogenetic tree of 125 Agrobacterium, Allorhizobium, Rhizobium and Neorhizobium species was inferred based on the concatenated protein alignments of 1165 single-copy genes. Ochrobactrum strain LBA8980 was used as an outgroup. The tree’s branch colors are used in the color bars of the heatmaps (Fig. 4, Figs. S3, S4 and S5) and in the replicon sizes bar chart (Fig. S6) to indicate corresponding genomes. NB, not every clade contains equally closely related bacterial species. The R. leguminosarum/R. indicum clade consists of rather closely related species (short branch lengths), in contrast to the orange and purple clades which have rather long branch lengths, but were not further subdivided in order to keep a clear view. The red clade, besides all Agrobacterium species, contains a number of Rhizobium species. This is not unexpected, since of these, Rhizobium pusense and Rhizobium oryzihabitans harbor (like A. tumefaciens and A. fabrum) a linear chromosome and R. pusense has also been named Agrobacterium genomospecies G2. Rhizobium sp. NIBRBAC000502774 shows a strange pattern in the heatmaps (Fig. 3, Figs. S3, S4 and S5). Detailed investigation of this genome assembly was beyond the scope of this study, but in the genome taxonomy database (GTDB) it is listed as an Agrobacterium species, with a CheckM completeness score of only 84.09%. [file 12866_2021_2358_MOESM2_ESM.pdf]

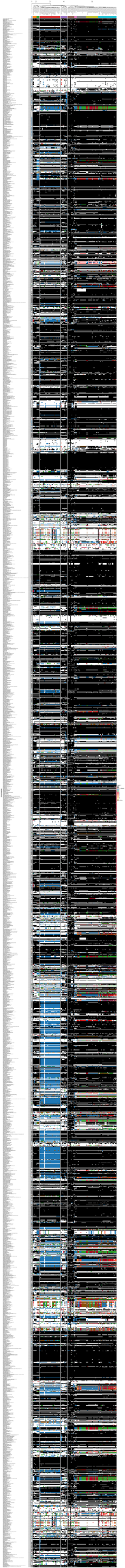

Supplement: Supplementary file 4 — Additional file 4: Figure S3. Heatmap showing conservation of AB2/73 chromosomal genes across Rhizobiaceae genomes. This figure is based on the same data as Fig. 4. However, in this larger version, rows were not clustered by similarity but ordered by the location of the genes in the AB2/73 chromosome. [file 12866_2021_2358_MOESM4_ESM.pdf]

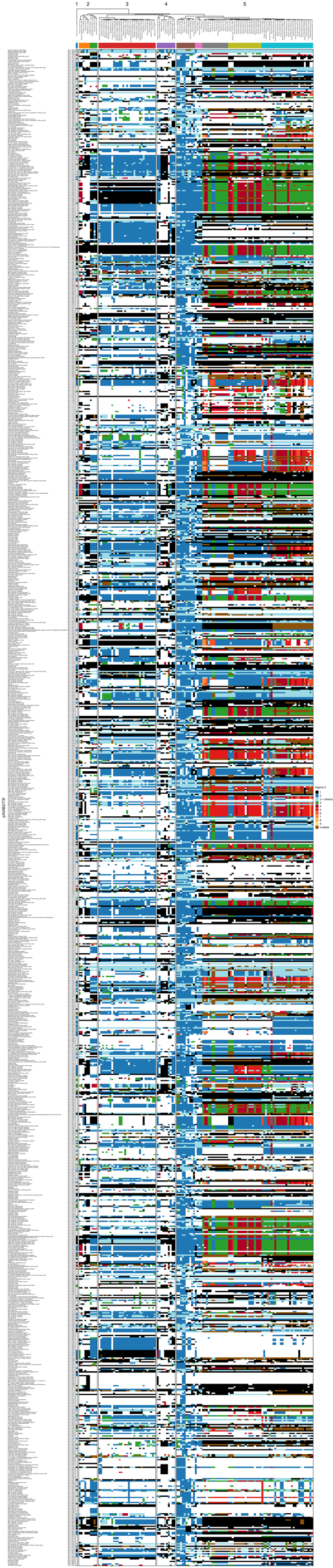

Supplement: Supplementary file 5 — Additional file 5: Figure S4. Heatmap showing conservation of pAtAB2/73f genes across Rhizobiaceae genomes. This figure is based on the same data as Fig. 4. However, in this larger version, rows were not clustered by similarity but ordered by the genes’ location in pAtAB2/73f. [file 12866_2021_2358_MOESM5_ESM.pdf]

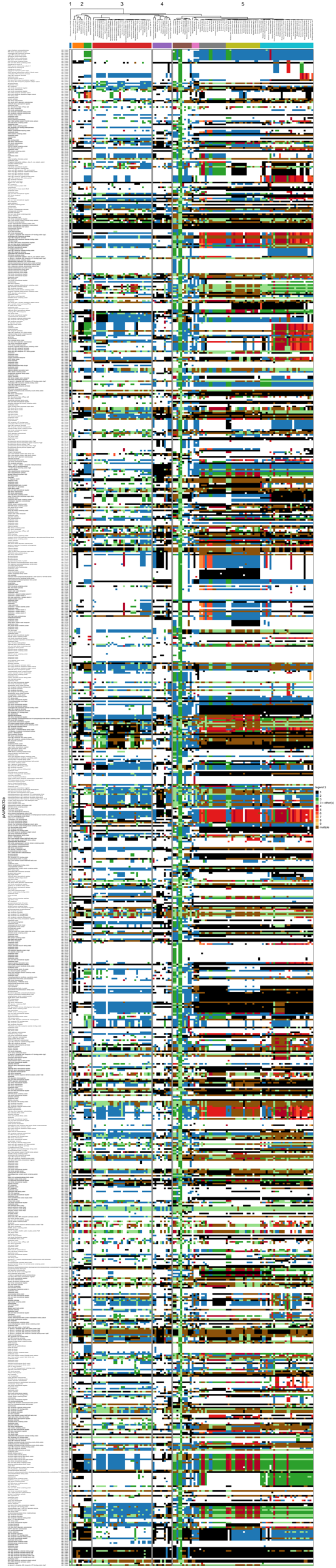

Supplement: Supplementary file 6 — Additional file 6: Figure S5. Heatmap showing conservation of pAtAB2/73e genes across Rhizobiaceae genomes. This figure is based on the same data as Fig. 4. However, in this larger version, rows were not clustered by similarity but ordered by the genes’ location in pAtAB2/73e. [file 12866_2021_2358_MOESM6_ESM.pdf]

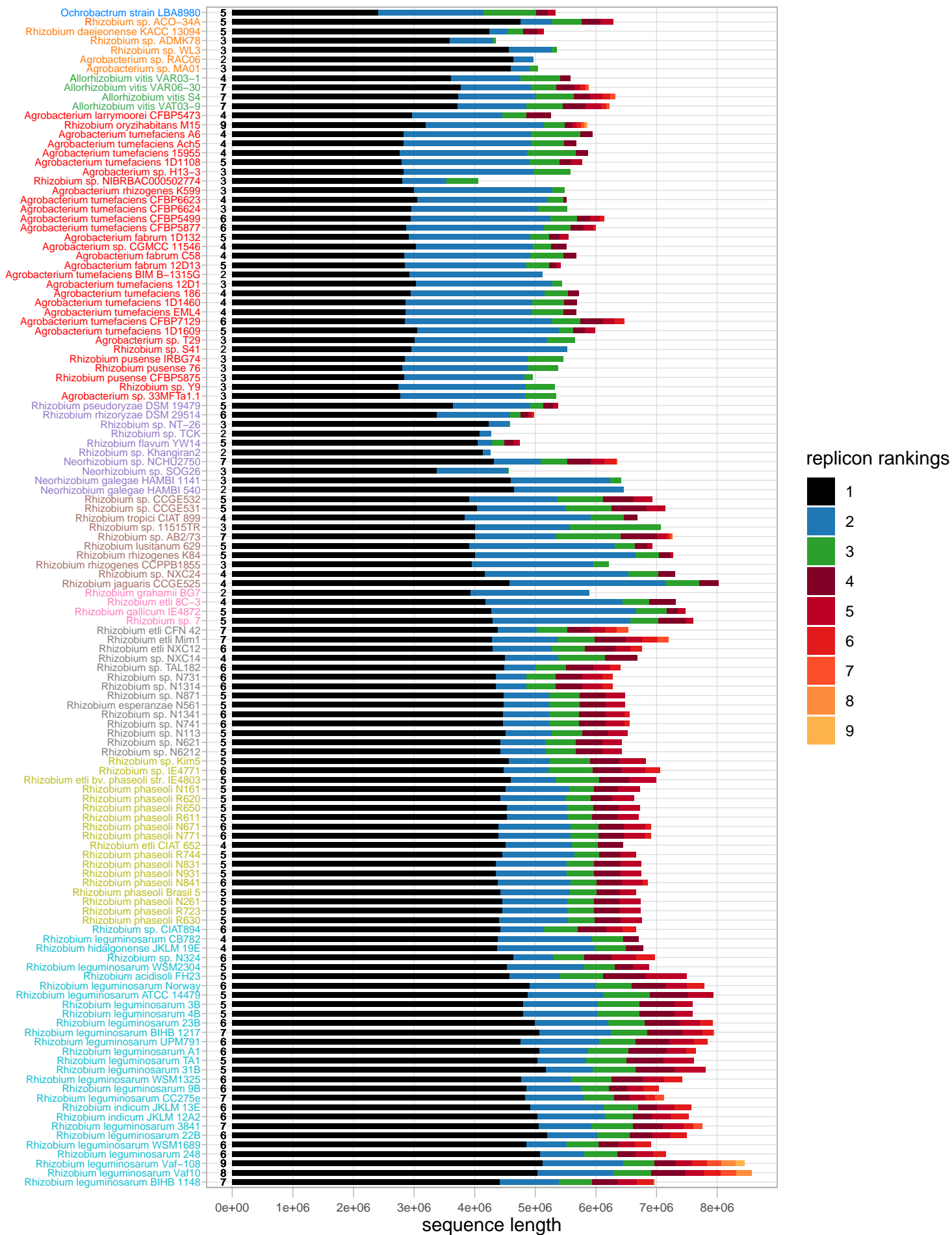

Supplement: Supplementary file 7 — Additional file 7: Figure S6. Stacked bar chart showing Agrobacterium/Rhizobium genome and replicon sizes. The text color and order of bars are as per the leaves of the species tree in Fig. S2. The replicon rank colors correspond to those used in the heatmaps. [file 12866_2021_2358_MOESM7_ESM.pdf]

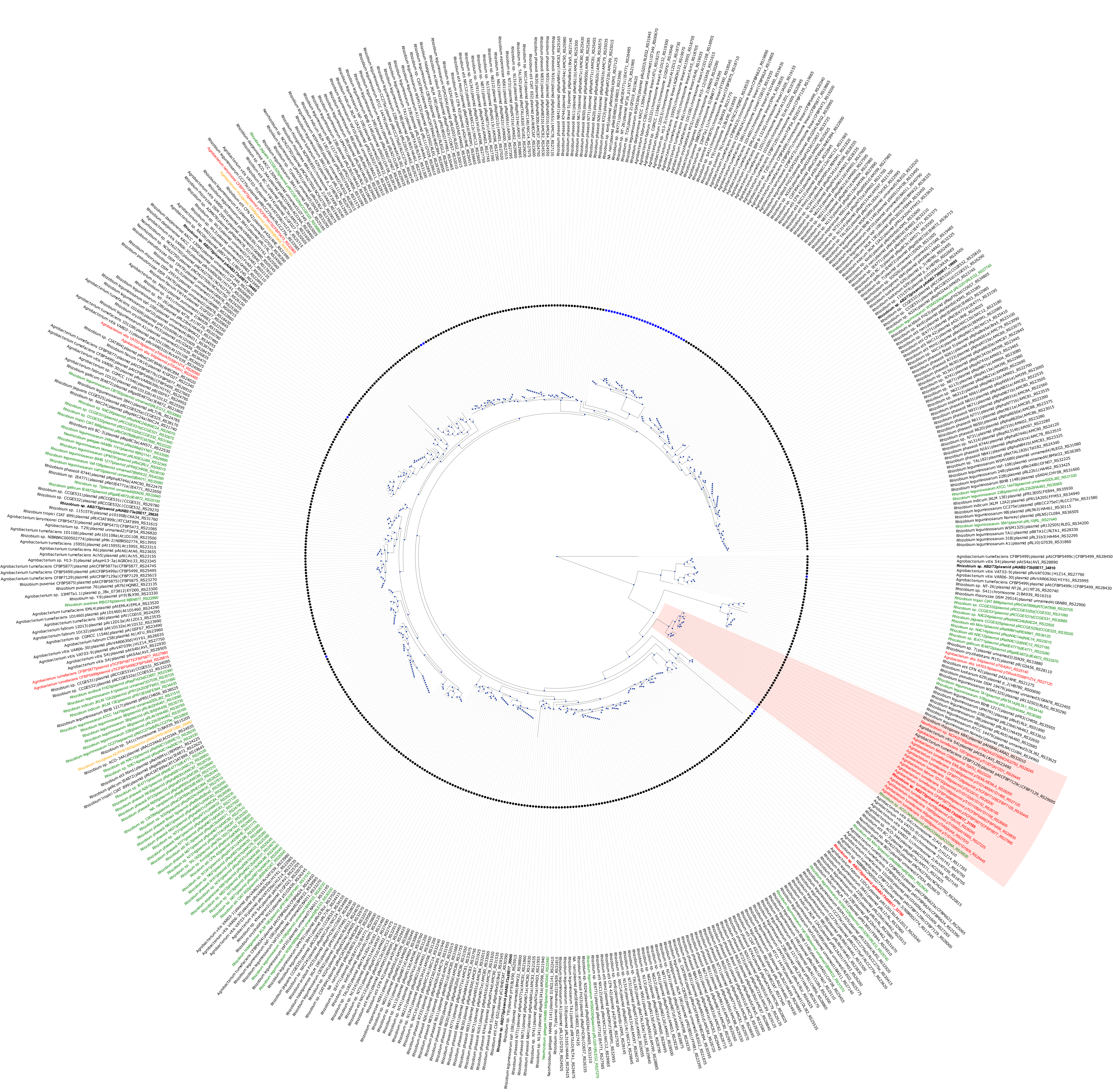

Supplement: Supplementary file 8 — Additional file 8: Figure S7. RepC phylogenetic tree. A maximum likelihood phylogenetic tree was constructed from the RepC protein sequences from 125 Rhizobium and Agrobacterium genomes. The resulting tree was midpoint rooted. The scale bar shows the number of amino acid changes per site. The tree was annotated with protein descriptions written as organism|replicon name|locus tag. AB2/73 proteins are printed in bold. Ti plasmid proteins are shown in red text, Ri plasmid proteins in orange text and Sym plasmid sequences in green. The black leaf nodes represent plasmid encoded proteins, whereas chromosomal proteins have blue leaf nodes (using the genome assemblies annotation as ‘chromosome’ or ‘plasmid’). Most (but not all) Ti plasmid RepC proteins are clustered together in a group indicated with red background. pTiAB2/73 harbors two repC genes. RepC encoded by the gene with locus tag I8E17_31105 (of the repB’-repC operon) lies in the group with the Ti plasmid RepC proteins, whereas the other RepC (of the repABC operon) groups with RepC proteins of some other non-Ti plasmids. RepC encoded by plasmids pAtAB2/73e and pAtAB2/73f cluster with RepC proteins from replicons of genomes to which AB2/73 is most related according to the species tree. [file 12866_2021_2358_MOESM8_ESM.pdf]

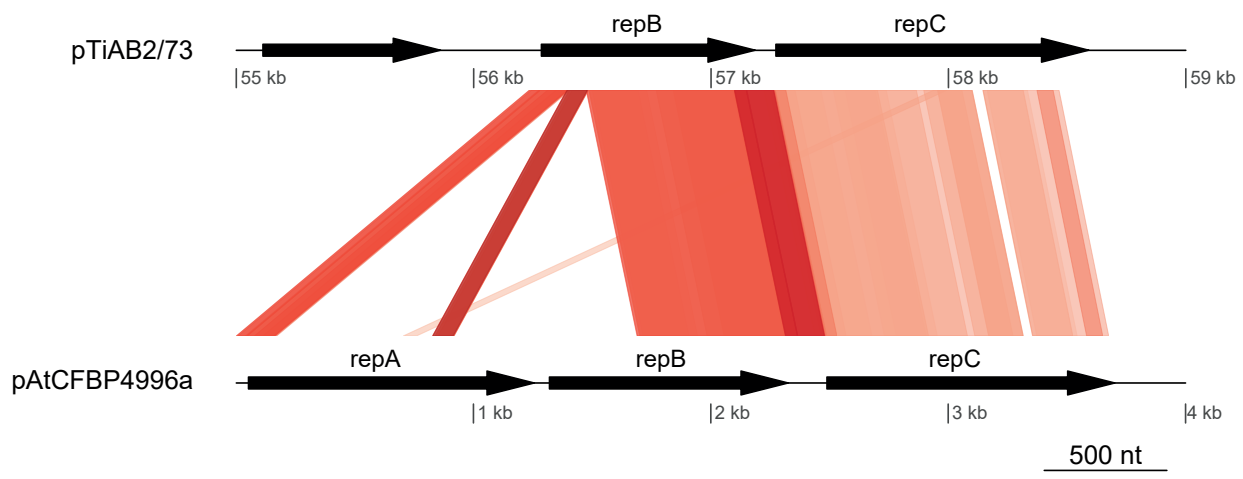

Supplement: Supplementary file 9 — Additional file 9: Figure S8. pTiAB2/73 repB of the repBC operon is truncated. A comparison is shown of pTiAB2/73 rep genes (locus tags I8E17_31100, I8E17_31105) with the repABC operon of plasmid pAtCFBP4996a. The red ribbons show tBLASTx hits (e < 0.001), with color intensity indicating the degree of sequence identity (darker = higher similarity). The C-terminal part of I8E17_31100 (“repB”) is conserved, yet the N-terminus shows similarity to the N-terminus and an internal part of the repA gene of pAtCFBP4996a. The gene I8E17_31100 thus appears to be a truncated repB fused to fragments of repA. [file 12866_2021_2358_MOESM9_ESM.pdf]

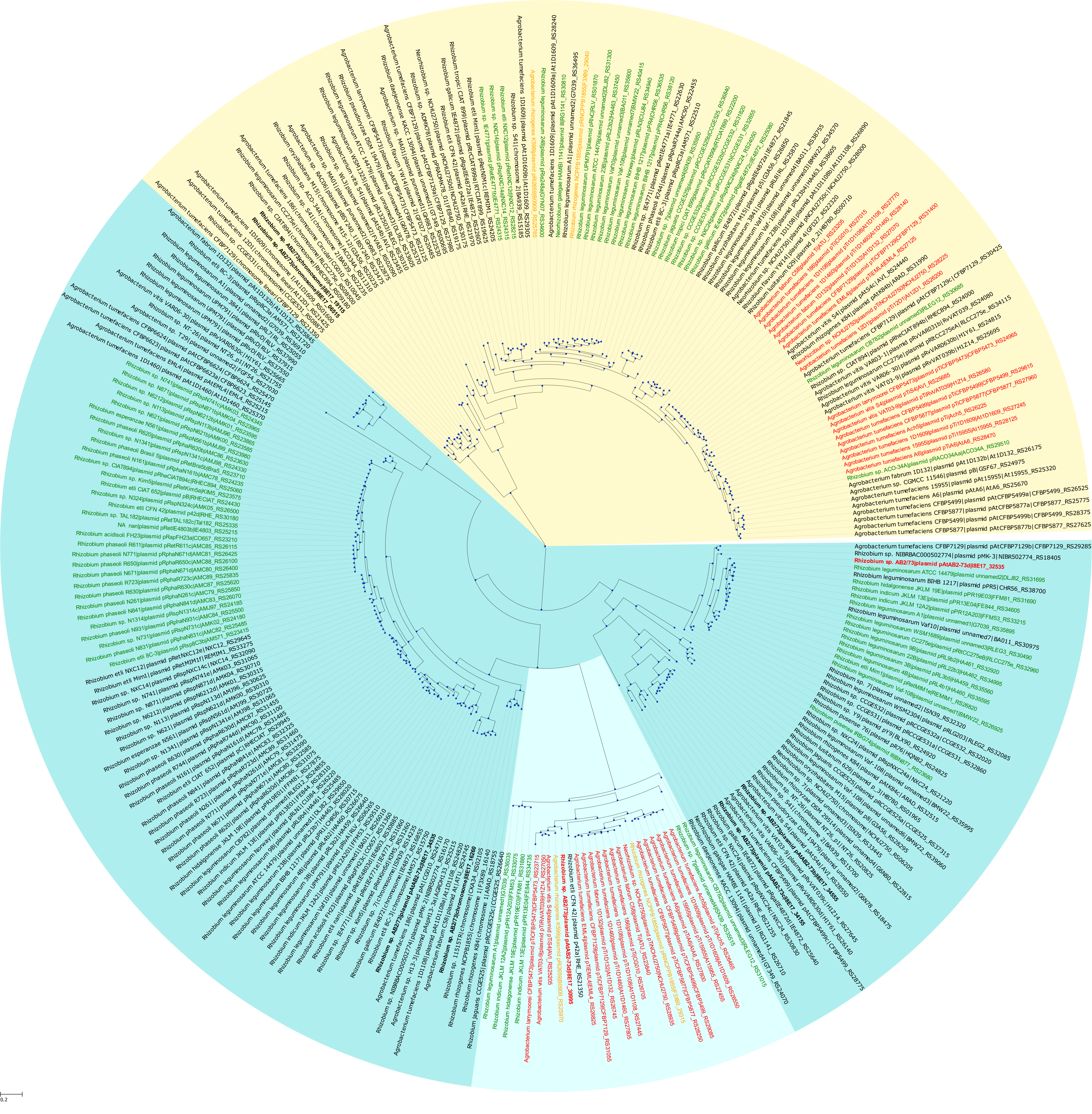

Supplement: Supplementary file 10 — Additional file 10: Figure S9. VirB11/TrbB phylogenetic tree. A maximum likelihood phylogenetic tree was constructed based on VirB11/TrbB protein sequences from 125 Rhizobium and Agrobacterium genomes. The tree was midpoint rooted. The scale bar shows the number of amino acid changes per site. The tree was annotated with protein descriptions written as organism|replicon name|locus tag. Ti plasmid proteins are shown in red text, Ri plasmid proteins in orange text and Sym plasmid sequences in green. AB2/73 proteins are printed in bold. TrbB sequences form a separate clade (the part with the yellow background). Generally, Ti and Ri plasmids have a trb region, so the TrbB clade includes Ti and Ri plasmid sequences, however a pTiAB2/73 TrbB homolog is lacking. The VirB11 sequences from the vir regions of Ti and Ri plasmids also cluster together (clade with very light blue background), including a pTiAB2/73 protein (locus tag I8E17_30995). The rest of the proteins in the tree (darker blue background) are also named ‘VirB11’ but these are not derived from Ti plasmid virB operons. The only exception is the second pTiAB2/73 VirB11 homolog. The gene encoding this protein (locus tag I8E17_32535) is located in the non-Ti-like part of pTiAB2/73. This protein clusters with sequences mostly encoded by Rhizobium (indicum, leguminosarum, hidalgonense and etli) Sym plasmids. Some of these Sym plasmids, like pTiAB2/73, have a second VirB11 homolog which is rather similar to vir region-containing VirB11 (green names in very light blue background). The similarity of pTiAB2/73 vir region virB genes and the second set of virB-like genes to two such Sym plasmids, as well as an example of a plasmid which only shows similarity of the virB-like operon, can be seen in Fig. 5 (rings pPR19E03, pPR12A203 and pAtCFBP7129b, respectively). [file 12866_2021_2358_MOESM10_ESM.pdf]

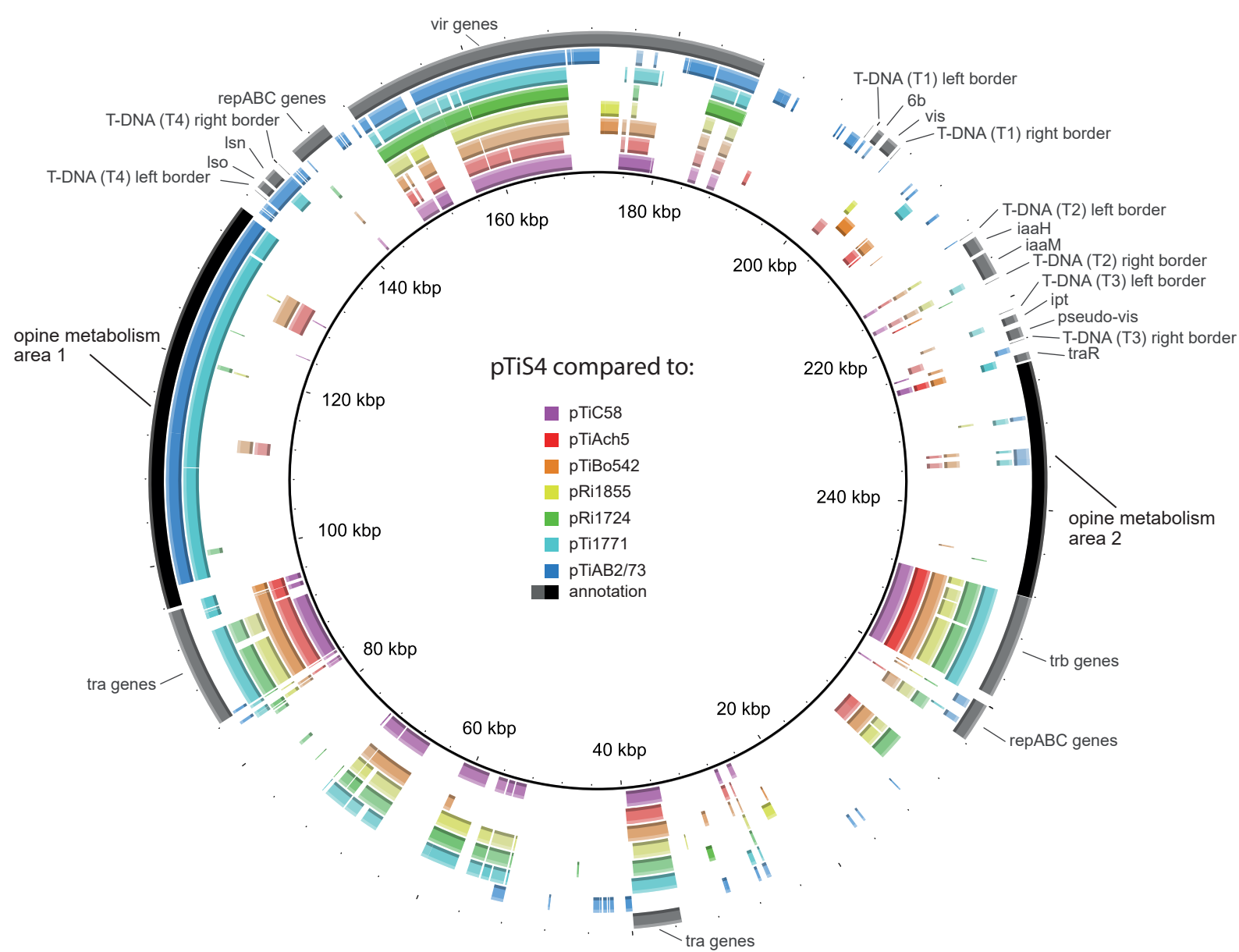

Supplement: Supplementary file 12 — Additional file 12: Figure S10. Ridéopine metabolism region presumably conserved across pTiS4, pTiAB2/73 and pTi1771. The sequence of pTiS4 (accession NC_011982) is compared to various Ti and Ri plasmid sequences. BLASTn hits are shown in concentric rings, from inner to outer ring: nopaline Ti plasmid pTiC58 (accession NC_003065), octopine Ti plasmid pTiAch5 (accession NZ_CP007228), agropine/succinamopine Ti plasmid pTiBo542 (accession NC_010929), agropine Ri plasmid pRi1855 (accession CP044124), mikimopine Ri plasmid pRi1724 (accession NC_002575), Ti plasmid pTi1771 (unknown opine type, unpublished, A. vitis strain NCPPB 1771) and pTiAB2/73 (accession CP067074). The DNA region adjacent to pTiS4 T-DNA region 4, and the nopaline-like opine synthase gene within T-DNA4 (lsn), are conserved (only) in pTiAB2/73 and pTi1771. Since A. vitis S4 produces the opines vitopine and ridéopine, and since the T-DNA regions 1-3 harbour either vitopine synthase or no opine synthase, the lsn gene in T-DNA4 likely encodes a ridéopine synthase, and the adjacent region likely harbors the genes required for ridéopine import and catabolism. [file 12866_2021_2358_MOESM12_ESM.pdf]

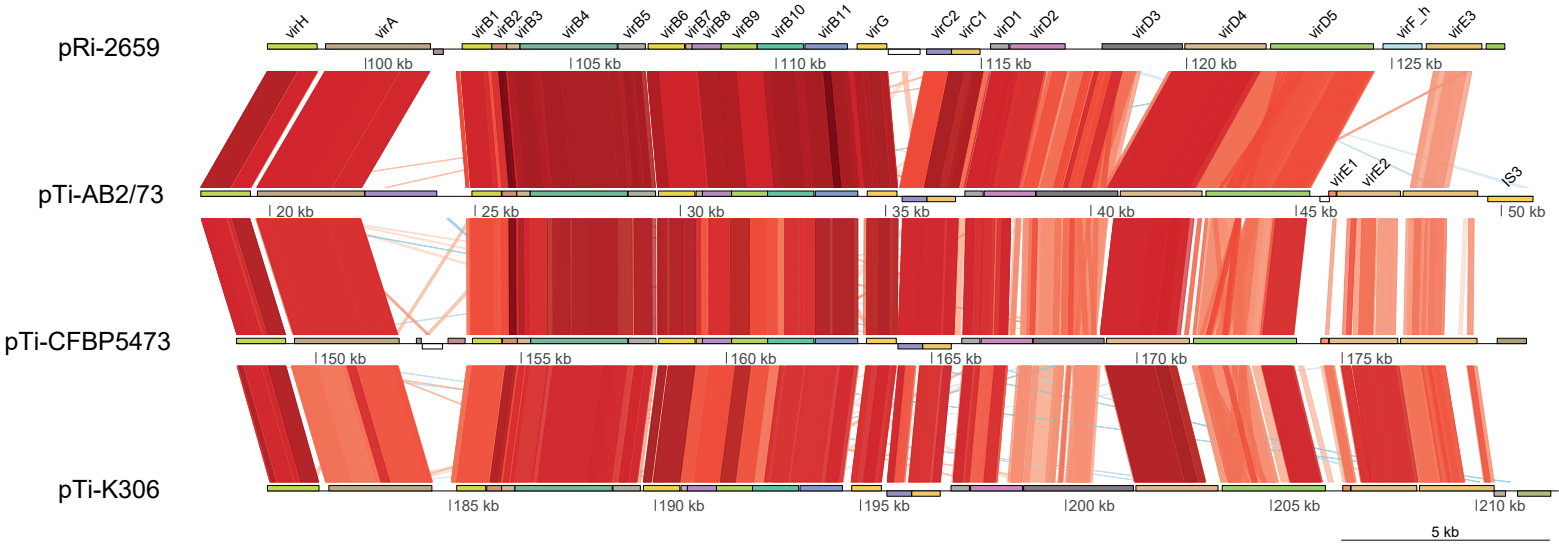

Supplement: Supplementary file 16 — Additional file 16: Figure S14. Comparison between of a number of Ti plasmid vir regions. Comparison between the vir region of pTiAB2/73 with those of pRi2659 (cucumopine Ri plasmid), pTiCFBP5473 (Ti plasmid from A. larrymoorei strain CFBP5473) and pTiK306 (Ti plasmid from A. vitis strain K306). The tBLASTx hits (e < 0.001) are shown in red, with darker bands indicating higher degrees of similarity. Most vir genes are well conserved, but virD3 and virD5, and virE1, virE2 and virE3 only to a lesser extent. [file 12866_2021_2358_MOESM16_ESM.pdf]
