# Supplementary material for: Complete genomic sequence and phylogenomics analysis of Agrobacterium strain AB2/73: a new Rhizobium species with a unique mega-Ti plasmid
Source: BMC Microbiol. 2021 Oct 28;21:295. doi: 10.1186/s12866-021-02358-0 (PMC8554961; doi:10.1186/s12866-021-02358-0)
Supplement: Supplementary file 11 — Additional file 11: Table S2. T-DNA border sequences found in pTiAB2/73. [file 12866_2021_2358_MOESM11_ESM.docx]

|  | Left border | Right border |
| --- | --- | --- |
| consensus | NGGCAGGATNTATNNNNNTGTAANN | TGNCAGGATNTATNNNNNNGTNNNN |
| T-DNA 1 | TGGCAGGATATATTGGTGTGTAAAC | TGGCAGGATGTATCAGATTGTAGTG |
| T-DNA 2 | CGGCAGGATATATTGCAACGTAAAA | GGGCAGGATTTATCGTTATGTCATG |

**Table S2. pTiAB2/73 T-DNA border sequences**
