# Supplementary material for: Complete genomic sequence and phylogenomics analysis of Agrobacterium strain AB2/73: a new Rhizobium species with a unique mega-Ti plasmid
Source: BMC Microbiol. 2021 Oct 28;21:295. doi: 10.1186/s12866-021-02358-0 (PMC8554961; doi:10.1186/s12866-021-02358-0)
Supplement: Supplementary file 13 — Additional file 13: Figure S11. IaaH/Tms2-like protein from pTiAB2/73 is more similar to non-T-DNA sequences than to T-DNA-encoded IaaH. Alignment of Indoleacetamide hydrolase/amidase sequences from pTiAB2/73, from two Rhizobiaceae family members (Rhizobium tumorigenes and Allorhizobium vitis, 87% identical), two betaproteobacterial amidases (Dickeya dianthicola, Trinickia symbiotica, 63% identical) and iaaH/tms2 from the T-DNAs of the Ti plasmids of A. tumefaciens strains C58, Ach5 (LBA4213) and S4 (32-34% identical). Residues identical in the majority of sequences are shown with black shading, residues similar in the majority of sequences are shown with grey shading. [file 12866_2021_2358_MOESM13_ESM.pdf]

```
AB2/73|I8E17_31255                       1 MFVRSTLEEICNQIQAGEISVLEIREATLAAMLRNSQLNCFIHDS-GVEQSFAKP--DAACVDAPLFGIPVSFKDNICVE  77
Rhizobium_tumorigenes|WP_111220954.1     1 MFATSNIEKICHQIHARETSVLEIRESTLAAMLRHSQLNCFIQER-GIEQSFTKP--DAACVDAPLFGIPVSFKDNICVE  77
Allorhizobium_vitis|WP_156634936.1       1 MFATSNIEKICHQIHARETSVLEIRESTLAAMLRHSQLNCFIQER-GIEQSFTKP--DAACVDAPLFGIPVSFKDNICVE  77
Dickeya_dianthicola|WP_161142954.1       1 MHSEYTLESLCSDLRRGTVSAAEIRQRALNIEAKNSQLNCFIRSSGDAEEHFAEA--DDSQKGAPLYGIPVSFKDNICVA  78
Trinickia_symbiotica|WP_102607270.1      1 MYSQRTLESLCNDLRNDALSAAEIRGRALSAEANLTKLNCFIRGR-DAEMHFGEA--NKLLKGAPLYGIPVSFKDNIYVK  77
C58(T-DNA)|Atu6010                       1 MVPITSLAQTLERLRRKDYSCLELVETLIARCQAAKPLNALLATDWDGLRRSAKKIDRHGNAGLGLCGIPLCFKANIATG  80
Ach5(T-DNA)|X971_RS25515                 1 MVAITSLAQSLEHLKRKDYSCLELVETLIARCEAAKSLNALLATDWDGLRRSAKKIDRHGNAGVGLCGIPLCFKANIATG  80
S4(T-DNA)|AVI_RS25485                    1 MVEISSISQTLRALRRKQYSCRDLVESLVSRSESATHLNAFAATDWLHLRNEADRVDRNGSGGVGLMGIPLCFKANIATG  80

AB2/73|I8E17_31255                      78 GQPVTVGTSAMAACIAPRDAKVVRNLKVLGAVVSGKNNMHELSFGVTSANIHWGTVGNPVAPGYCAGGSSGGAAAVAAG 157
Rhizobium_tumorigenes|WP_111220954.1    78 GQPVTVGTSAMAACIAPRDAEIVRQLKALGAVVSGKNNMHELSFGITSVNAQWGTVGNPAAPGYCAGGSSGGAAAVAAG 157
Allorhizobium_vitis|WP_156634936.1      78 GQPVTVGTSAMAACIAPRDAEIVRQLKALGAVVSGKNNMHELSFGITSVNAQWGTVGNPAAPGYCAGGSSGGAAAVAAG 157
Dickeya_dianthicola|WP_161142954.1      79 GLPVTVGTQGMADCIATHDAVIVRKLKSLGAVVAGKNNMNELCFGVTSIEPYWGVVENPSAPGYSVGGSSSGCAAAVAAG 158
Trinickia_symbiotica|WP_102607270.1     78 GLPVTAGTPGMADCIAPHDASIVSKLKSLGAVVAGKNNMHELSFGITSVNPRWGTVGNPVAPGYLAGGSSGGCAAAVAAN 157
C58(T-DNA)|Atu6010                      81 IFPTSAATPALINHLPKIPSRVAERLFSAGALPGASGNMHELSFGITSNNYATGAVRNPWNPSLIPGGSSGGVAAAVASR 160
Ach5(T-DNA)|X971_RS25515                81 VFPTSAATPALINHLPKIPSRVAERLFSAGALPGASGNMHELSFGITSNNYATGAVRNPWNPDLIPGGSSGGVAAAVASR 160
S4(T-DNA)|AVI_RS25485                   81 IFPTSAGTQGLLRHKPAIPAKIVERLHSAGALIGASGNMHELSFGITNDNKTFGPARNPWNQALISGGSSGGVAVSVAAN 160

AB2/73|I8E17_31255                     158 IVLVAVGTDTGGSVRIPASFCGIAGFRPTSGRWSSSGIIPVSGTKDSPGLLTRNAADALFVYKLLSPDEPATAA-DTSPL 236
Rhizobium_tumorigenes|WP_111220954.1   158 IVLLAVGTDTGGSVRIPASFCGITGFRPTSGRWSSSGIIPVSRTKDSPGLLTRSAADALFVYSHLSSDEPMTAP-DKSPL 236
Allorhizobium_vitis|WP_156634936.1     158 IVLLAVGTDTGGSVRIPASFCGITGFRPTSGRWSSSGIIPVSRTKDSPGLLTRSAADALFVYSHLSSDEPMTAP-DKSPL 236
Dickeya_dianthicola|WP_161142954.1     159 IVPIAIGTDTGGSARIPASFCGITGFRPTSGHWSSSGIIPVSHTKDSPGLMTRTASDAHFLYTQLSSDDVSTAEQGTFPC 238
Trinickia_symbiotica|WP_102607270.1    158 IVPVAIGTDTGGSVRIPAAFCGITGFRPTSGRWSSAGIIPVSRTKDSPGLLTRTARDARFLYELLSRDGRLPTEHEGSRC 237
C58(T-DNA)|Atu6010                     161 LMLGGIGTDTGASVRLPAALCGVVGFRPTLARYPRDRIIPVSPTRDTAGIIAQCVADVIILDQVISGRSAKISPMPLKGL 240
Ach5(T-DNA)|X971_RS25515               161 LMLGGIGTDTGASVRLPAALCGVVGFRPTLGRYPGDRIIPVSPTRDTPGIIAQCVADVVILDRIISGTPERIPPVPLKGL 240
S4(T-DNA)|AVI_RS25485                  161 LMLGGIGTDTGASVRLPAALCGVVGFRPTFGNYPTDGIVPVSPSRDTPGLIVRSVEDAVLLDRIIRNK-CPTQNMSLKGL 239

AB2/73|I8E17_31255                     237 RIGLPSS-LWTGLDVDVKSVCRAAIDSLKSVGHQCVEVDDASILELSQTITFTVPLYEFFLDFPRALVSLGWEDKISEVF 315
Rhizobium_tumorigenes|WP_111220954.1   237 RIGLPSS-LWTGLDADVKSVCRAAIDSLKSVEHQCVEVDDTSILELSRTITFTVPLYEFFLDFPRTLVSLGWEEKISEVF 315
Allorhizobium_vitis|WP_156634936.1     237 RIGLPSS-LWTGLDADVKSVCRAAIDSLKSVEHQCVEVDDTSILELSRTITFTVPLYEFFLDFPRTLVSLGWEEKISEVF 315
Dickeya_dianthicola|WP_161142954.1     239 RIGLPLS-MWSELDDDVMTHCRHAINQLVLAGFECVDVDDAAVFSLNQTITFTLPIYEFFVDFPRTLLSLGWENRITAVF 317
Trinickia_symbiotica|WP_102607270.1    238 RIGLPAS-MWTDLDDDVGKYCRHAIKRLTRAGFECIEVNDEIASTLNAPITFTVPLYEFFIDFPRALFSVGWEKNIKTVF 316
C58(T-DNA)|Atu6010                     241 RIGLPTTYFYDDLDADVAFAAETTIRLLANRGVTFVEADIPHLEELNSGASLPIALYEFPHALKKYLDDFVGTVSFSDVI 320
Ach5(T-DNA)|X971_RS25515               241 RIGLPTTYFYDDLDADVALAAETTIRLLANKGVTFVEANIPHLDELNKGASFPVALYEFPHALKQYLDDFVKTVSFSDVI 320
S4(T-DNA)|AVI_RS25485                  240 RLGLPRSHFFDNLEPHVAAASERAIRRLATNQMTFVEADIPNVAELTRKVSLPVAIYEFPRALMAYLSFHGIGNTFDELI 319

AB2/73|I8E17_31255                     316 ENIRDKNVCSLIHTHLGGGLITPAHYADAISNV-GRLRRKINALFNFGDIDLLAYPTVP-------QAVPLVSEAAHPDI 387
Rhizobium_tumorigenes|WP_111220954.1   316 EDIRDENVRGIIHAHLGGGLITPANYAEAISNV-GRLRRKINALFDLGDIDLLAYPTVP-------QAVPLVSEAAHPDI 387
Allorhizobium_vitis|WP_156634936.1     316 EDIRDENVRGIIHAHLGGGLITPANYAEAISNV-GRLRRKINALFDLDDIDLLAYPTVP-------QAVPLVSEAAHPDI 387
Dickeya_dianthicola|WP_161142954.1     318 DNISDKNVRNIIHEYLGGGRISPADYVSAVRNI-GRLRLDMDTLFSTYGIDMLVYPTVP-------RQVPLLSHAGRPEL 389
Trinickia_symbiotica|WP_102607270.1    317 DNIGDDDVHKVVHAHLSGRLICPADYTSAMRNI-GRLREQMDALFCACGIDLLAYPTVP-------RNVPHLSEAGRPEL 388
C58(T-DNA)|Atu6010                     321 KGIRSPDVANIVSAQIDGHQISNDEYELARQSFRPRLQATYRNYFRLYQLDAILFPTAPLAAKAIGQESSVIHNGSMMNT 400
Ach5(T-DNA)|X971_RS25515               321 KGIRSPDVANIANAQIDGHQISKAEYELARHSFRPRLQATYRNYFKLNRLDAILFPTAPLVARPIGQDSSVIHNGTMLDT 400
S4(T-DNA)|AVI_RS25485                  320 QNIQDPQVYDLVQSQLSKGLISESVYRRALRCYKPRLKATYENYYSSNGLDAVLFPSVPLTAKPVGLQTTLVHNGVETDT 399

AB2/73|I8E17_31255                     388 FAKCIRNTDLASNAALPSITIPVA-PKGALPVGLSFDAAFGHDRYLLEAATRFEKVISLQVGSKKQLSRAERLEPNSG 464
Rhizobium_tumorigenes|WP_111220954.1   388 FAECIRNTDLASNAALPSITIPVA-PKGALPVGLSFDAACGKDRYLLEAATGLEKVISYK------------------ 446
Allorhizobium_vitis|WP_156634936.1     388 FAECIRNTDLASNAALPSITIPVA-PKGALPVGLSFDAACGKDRYLLEAATGLEKVISYK------------------ 446
Dickeya_dianthicola|WP_161142954.1     390 FAELIRNTDLASNAAMPSVTLPVA-PEGALPVGLSFDASRGRDGYLLSMAARIETIIKP------------------- 447
Trinickia_symbiotica|WP_102607270.1    389 FAEVIRNTDLASNAAMPSITLPVA-PADALPVGLSLDAARGRDRYLLAKATHIENIVTRHR----------------- 448
C58(T-DNA)|Atu6010                     401 FKIYVRNVDPSSNAGLPGLSLPACLTPDRLPVGMEIDGLAGSDHRLLAIGAALEKAINFPSFPDAFN----------- 467
Ach5(T-DNA)|X971_RS25515               401 FKIYVRNVDPSSNAGLPGLSIPVCLTPDRLPVGMEIDGLADSDQRLLAIGGALEEAIGFRYFAGLPN----------- 467
S4(T-DNA)|AVI_RS25485                  400 FGIFVRNLDPSSNIGLPSLSVPVSLTPDRLPVGIQIEGPFGSDDMVLAIGRAVEEMVKFGQEY--------------- 462
```
