# Supplementary material for: Complete genomic sequence and phylogenomics analysis of Agrobacterium strain AB2/73: a new Rhizobium species with a unique mega-Ti plasmid
Source: BMC Microbiol. 2021 Oct 28;21:295. doi: 10.1186/s12866-021-02358-0 (PMC8554961; doi:10.1186/s12866-021-02358-0)
Supplement: Supplementary file 14 — Additional file 14: Figure S12. IaaM/Tms1-like protein from pTiAB2/73 is more similar to non-T-DNA sequences than to T-DNA-encoded IaaM. Alignment of tryptophan 2-monooxygenase -like sequences from pTiAB2/73, from two Mesorhizobium species, from one Rhizobium species (~ 90% identical), one betaproteobacterial oxidoreductase (Dickeya chrysanthemi, 68% identical), and tryptophan 2-monooxygenases (iaaM/tms1) from the T-DNAs of the Ti plasmids of A. tumefaciens strains C58, Ach5 (LBA4213) and S4 (46-50% identical). Residues identical in the majority of sequences are shown with black shading, residues similar in the majority of sequences are shown with grey shading. [file 12866_2021_2358_MOESM14_ESM.pdf]

```
AB2/73|I8E17_31260                        1 --------------------------------MLNSPIP-----------------------------------------   7
Mesorhizobium_sp|TIL83536.1               1 --------------------------------MLNNTIA-----------------------------------------   7
Mesorhizobium_loti|WP_065005780.1         1 --------------------------------MLNNTIA-----------------------------------------   7
Rhizobium_tumorigenes|WP_111220955.1      1 --------------------------------MLNSTIP-----------------------------------------   7
Dickeya_chrysanthemi|WP_027713301.1       1 -------------------------------MSKYAAFL-----------------------------------------   8
C58(T-DNA)|Atu6011                        1 MSASALLDNQCDHFSTKMVDLIMVDKADELDRRVSDAFSEREASRGRRITQISGECSAGLACKRLADGRFPEI-------  73
Ach5(T-DNA)|X971_RS25520                  1 MSASPLLDNQCDHLPTKMVDLTMVDKADELDRRVSDAFLEREASRGRRITQISTECSAGLACKRLADGRFPEI-------  73
S4(T-DNA)|AVI_RS25490                     1 ---------------------------------MANFFYSRITNRSYSTKNLLNIEDKGRLKDELEKTRQTNICEICLHP  47

AB2/73|I8E17_31260                        8 --------------------------------------------------------------------------------   7
Mesorhizobium_sp|TIL83536.1               8 --------------------------------------------------------------------------------   7
Mesorhizobium_loti|WP_065005780.1         8 --------------------------------------------------------------------------------   7
Rhizobium_tumorigenes|WP_111220955.1      8 --------------------------------------------------------------------------------   7
Dickeya_chrysanthemi|WP_027713301.1       9 --------------------------------------------------------------------------------   8
C58(T-DNA)|Atu6011                       74 ---SAGEKVAALSAYIYVGKEILGRILESEPWARARVSGLVAIDLAPFCMDFSEAQLLQTLFLLSGKRCASSDLSHFVAI 150
Ach5(T-DNA)|X971_RS25520                 74 ---SAGGKVAVLSAYIYIGKEILGRILESKPWARATVSGLVAIDLAPFCMDFSEAQLIQALFLLSGKRCAPIDLSHFVAI 150
S4(T-DNA)|AVI_RS25490                    48 RGHRASVCRQILMGFAYTSKTVLEGLLSTMPHDDAPLGKIFVTDLPPYDEQVPQVLLMQAAALVTSYEYSFEDLAYFLVL 127

AB2/73|I8E17_31260                        8 ---------------------------------------------------TTSLPSIDLLYDYGSFLRLSDCEGGIGSV  36
Mesorhizobium_sp|TIL83536.1               8 ---------------------------------------------------RNSLPSIDLLYDYGPFLRLSESEGGIGSF  36
Mesorhizobium_loti|WP_065005780.1         8 ---------------------------------------------------RNSLPSIDLLYDYGPFLRLSESEGGIGSF  36
Rhizobium_tumorigenes|WP_111220955.1      8 ---------------------------------------------------VASLPSIDLLYDYGSFLRLRDFEGRIGSV  36
Dickeya_chrysanthemi|WP_027713301.1       9 ---------------------------------------------------ANSVPCVDLLYDYAPFLQCSEAEGRIGYF  37
C58(T-DNA)|Atu6011                      151 SISKTARSRTLQMPPYEKGTTKRVTGFTLTLEEAVPFDMVAYGRNLMLKASAGSFPTIDLLYDYRSFFDQCSDSGRIGFF 230
Ach5(T-DNA)|X971_RS25520                151 SISKTAGFRTLPMPLYENGTMKCVTGFTITLEGAVPFDMVAYGRNLMLKGSAGSFPTIDLLYDYRPFFDQCSDSGRIGFF 230
S4(T-DNA)|AVI_RS25490                   128 PLQMALMQKS----PSLGKDFPVISGYSITKDS--VHSPVAFGRNLMPRGVSCEFPQIDVLYDYRGFLEGGAFSEGVTSF 201

AB2/73|I8E17_31260                       37 AQNSLRPRIGIVGAGISGLVAATELLRAGITDLVLFEARD-RMGGRAWSQIFDPREPNLIAEMGAMRFPSSATCLFHYLD 115
Mesorhizobium_sp|TIL83536.1              37 AQNSGRPRVGIVGAGISGLVAATELLRAGITDIVLFEARD-RMGGRAWSQIFDPREPHLIAEMGAMRFPSSATCLFHYLD 115
Mesorhizobium_loti|WP_065005780.1        37 AQNSRRPRVGIVGAGISGLVAATELLRAGITDIVLFEARD-RMGGRAWSQIFDPREPHLIAEMGAMRFPSSATCLFHYLD 115
Rhizobium_tumorigenes|WP_111220955.1     37 AQNSRRPRVGIVGAGISGLVAATELLRAGVTDLVLFEARD-RMGGRAWSQIFDPLEPNLIAEMGAMRFPSSATCLFYYLD 115
Dickeya_chrysanthemi|WP_027713301.1      38 PPGVPTPRVAIVGAGISGLVAATELLRAGVKDITLFEARDRRVGGRVWSQTFDPRYPHLIAEMGAMRFPSSETCLFYYLN 117
C58(T-DNA)|Atu6011                      231 PEDVPKPKVAIIGAGISGLVVASELLHAGVDDVTIYEASD-RVGGKLWSHAFKD-APSVVAEMGAMRFPPAASCLFFFLE 308
Ach5(T-DNA)|X971_RS25520                231 PEDVPKPKVAVIGAGISGLVVANELLHAGVDDVTIYEASD-RVGGKLWSHAFRD-APSVVAEMGAMRFPPAAFCLFFFLE 308
S4(T-DNA)|AVI_RS25490                   202 PKETKKPKVAVIGAGISGLVSATLLLRNGIDDVTIFEAKN-VVGGRAHTHFFKG-EPSVCAELGAMRFPRSQACLFYLLE 279

AB2/73|I8E17_31260                      116 KFRIDTAASFPDPGIVDTEVHYRGERYLWQAGESPPPLFKRVYQGWQALINDGYVHEGIQLPAPAKITTLLRSRRFDEAR 195
Mesorhizobium_sp|TIL83536.1             116 KLRIDTAASFPDPGIVDTEVHYRGARHLWQAGESPPPLFRRVHQGWQALISDGYVHEGIQLPAPAKITTLLRSHRFDQAR 195
Mesorhizobium_loti|WP_065005780.1       116 KLRIDTAASFPDPGIVDTEVHYRGARHLWQAGESPPPLFRRVHQGWQALISDGYVHEGIQLPAPAKITTLLRSHRFDQAR 195
Rhizobium_tumorigenes|WP_111220955.1    116 KFRIDTAASFPDPGIVDTEIHYRGERYLWQAGGPPPPLFRRVDQGWQALINDGYVHEGIQLLAPAKITKLLRSRRFDEAR 195
Dickeya_chrysanthemi|WP_027713301.1     118 KLDIATTTSFPDPGVVDTELHYRGVRHIWSAGDPPPSLFSRVHEGWVALLNEGYLHNGVPLVAPRDITAMLKSHCFDQAR 197
C58(T-DNA)|Atu6011                      309 RYGLSSMRPFPNPGTVDTNLVYQGLRYMWKAGQQPPKLFHRVYSGWRAFLKDGFHEGDIVLASPVAITQALKSGDIRRAH 388
Ach5(T-DNA)|X971_RS25520                309 RYGLSSMRPFPNPGTVDTYLVYQGVQYMWKAGQLPPKLFHRVYNGWRAFLKDGFYERDIVLASPVAITQALKSGDIRWAH 388
S4(T-DNA)|AVI_RS25490                   280 YLGINAMTKFPNPGTVDTGLYYRGRSYNWKAHSLPPAIFNRVHKGWRTFLHAGFVDGVAAFASPFTLTECLRLRNYEFAS 359

AB2/73|I8E17_31260                      196 QAWQAWLDSFRDLSFYSALVTIFTGPRPPGRVPWKRPEDFELFGSLGIGSGGFLPVYQAAFTEILRLVINGYEDDQRLIM 275
Mesorhizobium_sp|TIL83536.1             196 DAWQAWLDSFRDISFYSALVTIFTGPQPPGRVPWKRPEDFELFGSLGIGSGGFLPVYQAAFTEMLRLVINGYEDDQRLIM 275
Mesorhizobium_loti|WP_065005780.1       196 DAWQAWLDSFRDISFYSALVTIFTGPQPPGRVPWKRPEDFELFGSLGIGSGGFLPVYQAAFTEILRLVINGYEDDQRMIM 275
Rhizobium_tumorigenes|WP_111220955.1    196 EAWQAWLDSFRDASFYSALVTIFTGPRPPGRVPWKRPEDFELFGSLGIGSGGFLPVYQAAFTEILRLVINGYEDDQRMII 275
Dickeya_chrysanthemi|WP_027713301.1     198 TAWQAWLDAFRDYSFYSALVTIFTSNTPPGSVPWCRPDDFELFGSLGIGSGGFLPVYQAGFTEILRLVINGYEDDQRLII 277
C58(T-DNA)|Atu6011                      389 DSWQTWLNRFGRESFSSAIERIFLGTHPPGETWSFPHDWDLFKLMGIGSGGFGPVFESGFIEILRLVINGYEENQRMCS 468
Ach5(T-DNA)|X971_RS25520                389 DSWQIWLNRFGRESFSSGIERIFLGTHPPGETWSFPHDWDLFKLMGIGSGGFGPVFESGFIEILRLVINGYEENQRMCP 468
S4(T-DNA)|AVI_RS25490                   360 SLWQKWLDAFSSETFSSGIERIFRGAHPPGEKWTRDVDMELFKELGVGSGGFGPVFGCGFIEILRLIVNGYEDNVMLLL 439

AB2/73|I8E17_31260                      276 GGISLLVKRLAEQEFHGVSLQQRVRNGHVSRIYKQDGQILLMGATGQVEPFDRVIVTTSNRAMELAHCLTADGTFLNNEV 355
Mesorhizobium_sp|TIL83536.1             276 GGISLLVKRLAEQELNGVSLRQRVRYGHVSRIYKQGGQILVTCAGGQVVPFDRVIVTTSNRAMELAHRLTADGTFLTNEV 355
Mesorhizobium_loti|WP_065005780.1       276 GGISLLVNRLAEQELNGVSLRQRVRYGHVSRIYKQGGQILVTCAAGQVVPFDRVIVTTSNRAMELAHRLTADGTFLTNEV 355
Rhizobium_tumorigenes|WP_111220955.1    276 GGISLLVKRFAEQEFHGLSLRQRVRNGHVSGISKQGGQILVTCSGGQVEPFDRVIVTTSNRTMELAHRLTTDETFLNNEV 355
Dickeya_chrysanthemi|WP_027713301.1     278 GGISTLAERLVSQKIGDTCLSERICFNEIKRIYKENGEIKLVSGKGQTYAFDRVIVTSSTRTMQIVHCLTGDETFLERDI 357
C58(T-DNA)|Atu6011                      469 EGISELPRRIATQVVNGVSVSQRIRHVQVRAIEKEKTKIKIRLKSGISELYDKVVVTSGLANIQLRHCLTCDTTIFRAPV 548
Ach5(T-DNA)|X971_RS25520                469 EGISELPRRIASEVVNGVSVSQRICHVQVRAIQKEKTKIKIRLKSGISELYDKVVVTSGLANIQLRHCLTCDTNIFQAPV 548
S4(T-DNA)|AVI_RS25490                   440 DGIEEIPRRLSQQKVGSYSIRDRIIHKEVKEIIRTESGISLAIGEGMHATFDRVIVTSGFTNIQLRHLLTNDDSFFSYDV 519

AB2/73|I8E17_31260                      356 LRAVRQTHLTGSSKLFMLTESKFWLKGVLPTTILSDGLARGVYCLDYQPDDPDGKGVVLLSYTWEDDANKMLSILDKKER 435
Mesorhizobium_sp|TIL83536.1             356 LRAVRETHLTGSSKLFMLTENKFWLKKGLPTTILSDGLARGVYCLDYQPDDPDGKGVVLLSYTWEDDANKMLSILDKKER 435
Mesorhizobium_loti|WP_065005780.1       356 LRAVRETHLTGSSKLFMLTENKFWLKKGTPTTILSDGLARGVYCLDYQPDDPDGKGVVLLSYTWEDDANKMLSILDKKER 435
Rhizobium_tumorigenes|WP_111220955.1    356 LRAVRRTHLTGSSKLFMLTENKFWLKEGLPTTILSDGLARGVYCLDYQPDKPDGKGVVLLSYTWEDDANKMLSILDKKER 435
Dickeya_chrysanthemi|WP_027713301.1     358 SRAVKETHLTGSSKLFMLTQNKFWLKHSMPATIQSDGFIRGVYCLDYEPDNPDGPGVVLLSYTWEDDAHKLLSIPDKKQR 437
C58(T-DNA)|Atu6011                      549 NQAVDNSHMTGSSKLFLLTERKFWLDHILPSCVLMDGIAKAVYCLDYEPQDPNGKGLVLISYTWEDDSHKLLAVPDKKER 628
Ach5(T-DNA)|X971_RS25520                549 NQAVDNSHMTGSSKLFLMTERKFWLDHILPSCVLMDGIAKAVYCLDYEPQDPNGKGLVLISYTWEDDSHKLLAVPDKKER 628
S4(T-DNA)|AVI_RS25490                   520 NQAIENSHMTGSSKLFVLTQNKFWKAEELPSCILTTGVAKAVYCLDYEPDKPSGKGLVLLSYTWEDDSHKLLTF-DKGER 598

AB2/73|I8E17_31260                      436 CRRLVDDLATISADFARHLVPANGDYERHVLQHDWLMDPYSIGAFKLNYPGEDIYSQQLFFQFTTAKRPEEDTGLYLAGC 515
Mesorhizobium_sp|TIL83536.1             436 CQRLVDDLATISPDFARHLVPANGDYERHVLQHDWLMDPYSVGAFKLNYPGEDIYSERLFFQFATAKRPEEDTGLYLAGC 515
Mesorhizobium_loti|WP_065005780.1       436 CQRLVDDLATISPDFARHLVPANGDYERHVLQHDWLMDPYSVGAFKLNYPGEDIYSERLFFQFATAKRPEEDTGLYLAGC 515
Rhizobium_tumorigenes|WP_111220955.1    436 CQRLVDDLATVCGDFARHLVPAKGDYERHVLQHDWLMDPYAVGAFKLNYPGEDIFSQRLFFQFATAKRPEEDTGLYLAGC 515
Dickeya_chrysanthemi|WP_027713301.1     438 CQYLVDDLAKIHPEFARHLIPADGDYERYVLHHDWLVDPYSAGAFKLNYPGEDVYSQRLFFQFKNANTPEKDTGLYLAGC 517
C58(T-DNA)|Atu6011                      629 FCLLRDAISRSFPAFAQHLVPACADYDQNVVQHDWLTDENAGGAFKLNRRGEDFYSEELFFQALDM---TNDTGVYLAGC 705
Ach5(T-DNA)|X971_RS25520                629 LCLLRDAISRSFPAFAQHLFPACADYDQNVIQHDWLTDENAGGAFKLNRRGEDFYSEELFFQALDT---ANDTGVYLAGC 705
S4(T-DNA)|AVI_RS25490                   599 FQILKRDLAKSYPRFADLLEPADGDYDNNIIQHDWILDPYAGGAFKLNRRCEDVYSKRLFFQPLRL-NGEPDGRVCLAGC 677

AB2/73|I8E17_31260                      516 GCSFTGGWVEGALQTGLNAACAVIRSCGGQLLEGNPIDEMTSAYRY---- 561
Mesorhizobium_sp|TIL83536.1             516 GCSFTGGWVEGAVQTGLNAACAVIRSSGGQLLSGNPIDSMNSAYRY---- 561
Mesorhizobium_loti|WP_065005780.1       516 GCSFTGGWVEGAVQTGLNAACAVIRSSGGQLLSGNPIDSMNSAYRY---- 561
Rhizobium_tumorigenes|WP_111220955.1    516 GCSFTGGWVEGAVQTGLNAACAVIRSCGGQLLQGNPIDEMDSAYRY---- 561
Dickeya_chrysanthemi|WP_027713301.1     518 GCSFTGGWIEGAMQTALNSACAVIRSSGGALLVGNPLDDMHSAYCY---- 563
C58(T-DNA)|Atu6011                      706 SCSFTGGWVEGAIQTACNAVCAIIHNCGGILAKDNPLEHSWKRYNYRNRN 755
Ach5(T-DNA)|X971_RS25520                706 SCSFTGGWVEGAIQTACNAVCAIIHNCGGILAKGNPLEHSWKRYNYRTRN 755
S4(T-DNA)|AVI_RS25490                   678 SCSFSGGWVEGAIQTACNAAMATIRDAGGLISGDNPLTNEFVNYHY---- 723
```
