# Supplementary material for: Complete genomic sequence and phylogenomics analysis of Agrobacterium strain AB2/73: a new Rhizobium species with a unique mega-Ti plasmid
Source: BMC Microbiol. 2021 Oct 28;21:295. doi: 10.1186/s12866-021-02358-0 (PMC8554961; doi:10.1186/s12866-021-02358-0)
Supplement: Supplementary file 15 — Additional file 15: Figure S13. pTiAB2/73 Ipt/Tzs-like protein shows strongest similarity to tzs-encoded isopentenyl transferases. Alignment of pTiAB2/73 tzs-like encoded protein to isopentenyl transferases from multiple Ri and Ti plasmids. Sequence identity versus pTiAB2/73 tzs-like varied from 44% (pTiS4 ipt) to 83% (pRi2659 tzs). Residues identical in the majority of sequences are shown with black shading, residues similar in the majority of sequences are shown with grey shading. [file 12866_2021_2358_MOESM15_ESM.pdf]

```
pTi-AB2/73|I8E17_31265   1 MLIYLIYGPTCSGKTDLAIQVARETGWPVIALDRVQCCPEIATGSGRPLASELQSTQRIYLDSRPLAAGVIEAEAAHRKL  80
pRi-2659|B0909_RS26265   1 MLIYLIYGPTCSGKTDLAVQVARETGWPVIALDRVQCCPQIATGSGRPLESELQSTERIYLDSRPLAEGVIDAEAAHRQL  80
pRi-1855|F3X89_29245     1 MLLYLIYGPTCSGKTDIAIQIAQKTGWPVVALDRVQCCPQIATGSGRPLPSELQSTRRIYLDSRRLTKGIIDAEGAHRRL  80
pTi-C58|Atu6164          1 MLLHLIYGPTCSGKTDMAIQIAQETGWPVVALDRVQCCPQIATGSGRPLESELQSTRRIYLDSRPLTEGILDAESAHRRL  80
pTi-S4|AVI_RS25515       1 MEAHLIFGPTSTGKTSVAIALAKRTGFPVIVLDRIQCYSQLSVGGGRPSAAEFQGTRRIYLIEGSLDEGVISAERAHECL  80
pTi-C58|Atu6012          1 MDLRLIFGPTCTGKTSTAVALAQQTGLPVLSLDRVQCCPQLSTGSGRPTVEELKGTSRLYLDDRPLVKGIIAAKQAHERL  80
pTi-Ach5|X971_RS25525    1 MDLHLIFGPTCTGKTTTAIALAQQTGLPVLSLDRVQCCPQLSTGSGRPTVEELKGTTRLYLDDRPLVEGIIAAKQAHHRL  80

pTi-AB2/73|I8E17_31265  81 ISEVENRKKSPGLILEGGSISLLNCMAKDPFWNAAFRWHVRRLRLATRDVFLPHARRRVTEMFAVKNDRPSLLEELVDLW 160
pRi-2659|B0909_RS26265  81 ISEVENRRTSPGLILEGGSISLLNCMATDTYWEADFRWHVRRLRLAAPDVFLPRARRRVTEMFAVKDERRSLLQELTDLW 160
pRi-1855|F3X89_29245    81 ILEVDWQESEEGLILEGGSVSLLNCMAKSPYWKSGFQWHVKRLRLGDSDAFLARAKQRVTEMFAIREDRPSLLEELAELW 160
pTi-C58|Atu6164         81 IFEVDWRKSEEGLILEGGSISLLNCMAKSPFWRSGFQWHVKRLRLGDSDAFLTRAKQRVAEMFAIREDRPSLLEELAELW 160
pTi-S4|AVI_RS25515      81 VAAVEAHKPEGGVILEGGSISLFKRMAQSSYWNCGFTWHVTRLHLGGEEIFLAAAKKRINQMMQPDEQGNSFLGELVSVW 160
pTi-C58|Atu6012         81 MGEVYNYEAHGGLILEGGSISLLKCMAQSSYWSADFRWHIIRHELADEETFMNVAKARVKQMLRPA-AGLSIIQELVDLW 159
pTi-Ach5|X971_RS25525   81 IEEVYNHEANGGLILEGGSTSLLNCMARNSYWSADFRWHIIRHKLPDQETFMKAAKARVKQMLHPA-AGHSIIQELVYLW 159

pTi-AB2/73|I8E17_31265 161 KQEALRPILEDIDGYRCAIRFAREHNIGISDLLRLDSTRQQALIAAIADEYFDHAQWQERDFP-DCGQHDGFRLSNPYAV 239
pRi-2659|B0909_RS26265 161 KQEALRPILEDIDGYRCAIRFAKEHDIGIRDLLRLDLTRQQALISAIADEYFDHAQWQERDFP-DWQEGGNVRLTPRVAS 239
pRi-1855|F3X89_29245   161 NYPATRPILEDIDGYRCAIRFARKHDLAINQLPDIDAERQQDLIEAIAKEYLEHAIMQERDFP-QWPEDGARQPVGPATL 239
pTi-C58|Atu6164        161 NYPAARPILEDIDGYRCAIRFARKHDLAISQLPNIDAGRHVELIEAIANEYLEHALSQERDFP-QWPEDGAGQPVCPVTL 239
pTi-S4|AVI_RS25515     161 KTTALRATLEGICGYRYAIEFAGKQGLEMDALTSLNRRQLEQLVHGMAHEYLCYARQQEQELP-LPSLAGG--EGPPFQ- 236
pTi-C58|Atu6012        160 KEPRLRPILKEIDGYRYAMLFASQNQITSDMLLQLDADMEDKLIHGIAQEYLIHARRQEQKFPRVNAAAYDGFEGHPFGM 239
pTi-Ach5|X971_RS25525  160 NEPRLRPILKEIDGYRYAMLFASQNQITADMLLQLDANMEGKLINGIAQEYFIHARQQEQKFPQVNAAAFDGFEGHPFGM 239

pTi-AB2/73|I8E17_31265 240 SSSTNEICYR 249
pRi-2659|B0909_RS26265 240 NAN------- 242
pRi-1855|F3X89_29245   240 MRIQ------ 243
pTi-C58|Atu6164        240 TRIR------ 243
pTi-S4|AVI_RS25515         ----------
pTi-C58|Atu6012        240 Y--------- 240
pTi-Ach5|X971_RS25525  240 Y--------- 240
```
